# Shift of human pathogen community composition and their potential human health risk after supply suspension in tap water

**DOI:** 10.1038/s41598-023-39225-z

**Published:** 2023-08-01

**Authors:** Shengnan Liu, Qisheng Li, Ruiming Jiang, Peng Liu, Xu-Xiang Zhang

**Affiliations:** 1 State Key Laboratory of Pollution Control and Resource Reuse, School of the Environment, Nanjing University, 163 Xianlin Road, Nanjing, 210023 China; 2 China Three Gorges Construction Engineering Corporation, Beijing, 100048 China

**Keywords:** Environmental impact, Natural hazards

## Abstract

Water supply suspension–restoration can occur frequently due to the overhauling of civil infrastructure in developing countries and the shutdown of commercial buildings during the pandemic. For comprehensive insights into the effects of water supply suspension–restoration, this study characterized the variations of the pathogen community composition of the tap water and their infection risk under different water supply scenarios. Metagenomic sequencing revealed a significant change of the human pathogen profiles, among which the most dominant pathogen changed from *Pseudomonas aeruginosa* (4.91%) to *Acinetobacter johnsonii* (0.59%). Furthermore, absolute quantification of pathogens by propidium-monoazide-qPCR revealed that the abundance of the three typical pathogens (*Pseudomonas aeruginosa*, *Mycobacterium avium* and *Salmonella* sp.) showed an increase of 2.44 log to 3.60 log immediately after water supply suspension–restoration and did not return to the normal level even after 2-h supply restoration, except for *Pseudomonas aeruginosa*. Quantitative microbial risk assessment suggested the infection risks of the three pathogens arising from direct utilization of tap water under stable water supply, including dermal exposure and oral intake, were all above the threshold of 10^−4^, and evidently increased after water supply suspension–restoration. This study warns us against the risk induced by the pathogens in tap water, especially after water supply suspension–restoration.

## Introduction

A variety of human microbial pathogens are present in drinking water and may be considered environmental contaminants that induce public health risk^1^. The urban drinking water distribution system (DWDS) is a transmission bridge between drinking water treatment plants and city dwellers^2^ and tap water (TW) is closely related to human living. Opportunistic pathogens are frequently detected in TW^3^, including *Legionella pneumophila*, *Mycobacterium avium* and *Pseudomonas aeruginosa*. Many countries or organizations have already included pathogens (such as *Salmonella* spp., *Legionella* spp. and *Campylobacter jejuni*) in drinking water safety guideline or standard^4,5^. Due to the large number of the population still living in water-scarce areas^6^, intermittent water supply is adopted in most low- and middle-income countries worldwide^7,8^, which is an important source of microbial infection in DWDS^9^. Besides, water supply suspension can also occur during the overhauling of civil infrastructure or the shutdown of commercial buildings during the pandemic^10^. During the water supply suspension, water quality undergoes serious deterioration and pathogenic bacteria can proliferate due to the consumption of residue chlorine^11^. What’s worse, the biofilm may detach from the pipes when the water flow in motion is suddenly forced to stop or start^12^, in which case the attached bacteria will enter the TW after the water supply restoration. Actually, the impact of pathogens in TW, especially after water supply suspension–restoration, on human health has become critical for risk control, which has not been well documented yet^13^.

For risk assessment, the detection methods of pathogens are of the essence^14^, both in terms of the coverage of diversity and the precision of absolute quantification. Traditional culture-dependent or molecular methods cannot cover all the pathogens in TW. Combined use of high throughput sequencing and metagenomic analysis is considered as a powerful tool to analyze the whole community of pathogens in the environments, including river^15^, sewage sludge^16^, human nasal cavity and feces^17^, which also serves as an effective and reliable approach for the comprehensive investigation of pathogens in clean environmental compartments, e.g. TW^18^. However, the relative abundance obtained from this method cannot be directly used for the assessment of the health risks induced by pathogens in TW, which relies on absolute quantification.

It is crucial to obtain the cell numbers of live pathogens in given volume of TW for precisely assessing their health risk with quantitative microbial risk assessment^19^ (QMRA), a method commonly employed in assessing health risk posed by waterborne pathogens^20^. Technically, quantitative real time polymerase chain reaction (qPCR) has been widely applied to absolutely quantify pathogens in water^21,22^, but cannot identify live or dead bacteria. Fortunately, propidium monoazide (PMA) is able to selectively combine with the naked DNA because of their nonpermeability for intact membrane cells among bacterial isolates and environmental samples^23,24^. After staining, DNA within dead cells and extracellular DNA would not be amplified during PCR due to the covalent link between dye and DNA molecule. PMA pretreatment of samples has been coupled with qPCR, namely PMA-qPCR, to trace live pathogens of concern in seawater^25^, agricultural produce^26^ and drinking water^27^.

This study aimed to investigate the composition, absolute abundance and health risk of pathogens in TW under stable water supply and suspended–restored water supply. Metagenome sequencing and bioinformatics analysis were conducted for comprehensive insights into the composition of the bacterial pathogens in TW. QMRA was employed to assess the health risks of the pathogens based on absolute quantification by PMA-qPCR. This study helps to extend our knowledge regarding the diversity and risks of pathogens in TW, which may facilitate the prevention and control of pathogen spread.

## Results

### Potential pathogens in tap water under stable water supply

**Figure 1:**
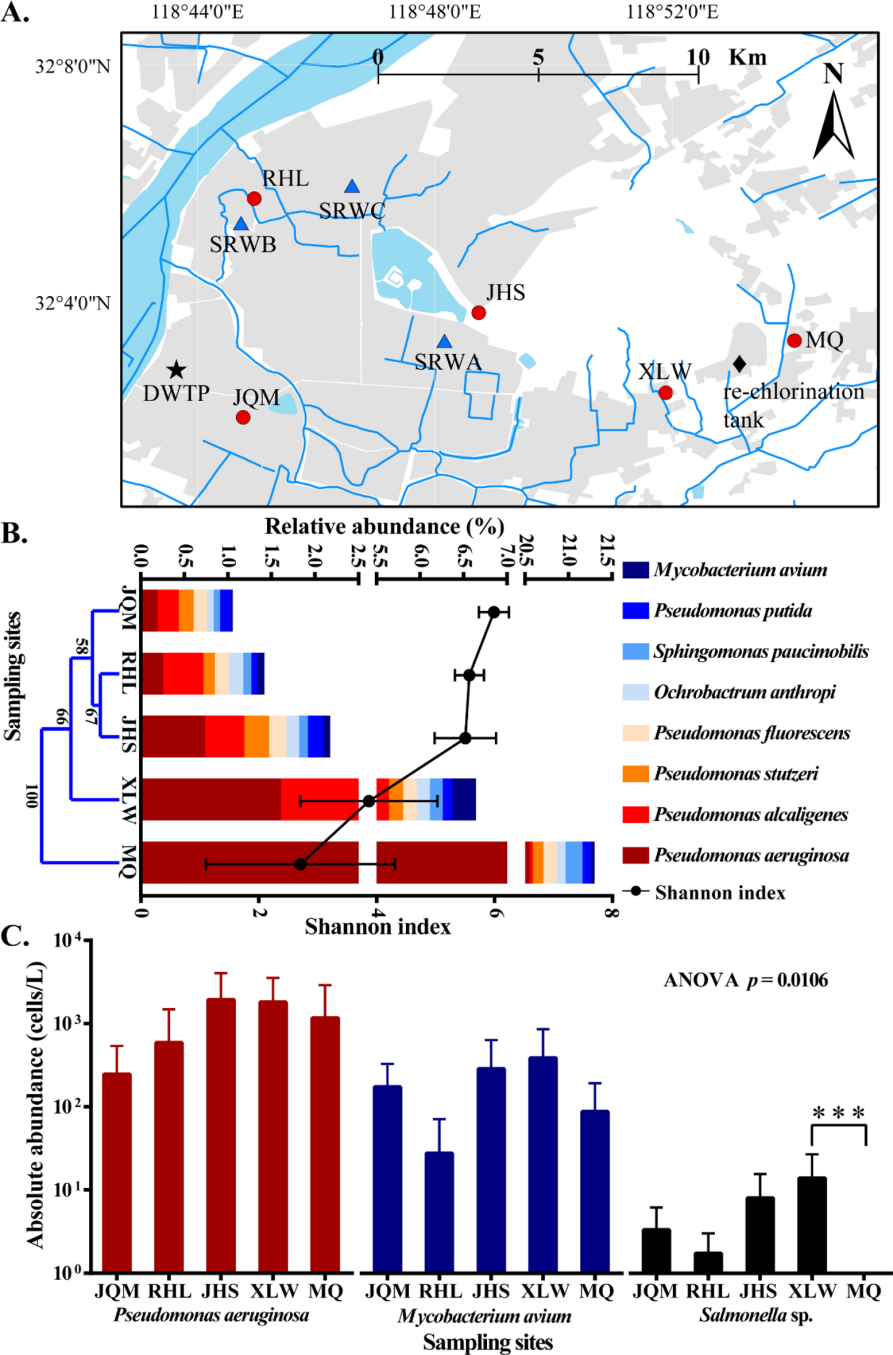
Geographic location of sampling sites, pathogen community composition and absolute abundance of the three typical pathogens in SW. (**A**) DWTP, re-chlorination tank, SW and suspension-restoration water (SRW) sampling locations are marked as black pentagram, black rhombus, red circle and blue triangle, respectively. (**B**) Relative abundance of abundant (mean value > 0.1%) pathogens at the different sites. Cluster analysis based on Bray–Curtis distance and Shannon index (mean ± standard error, n = 3) shows structural similarity and alpha diversity of pathogen community at the different sampling sites. (**C**) Absolute abundance (mean ± standard error, n = 10) of the three typical pathogens in SW at the different sites. *** means significant difference by *t*-test between sites XLW and MQ (*p* < 0.001).

We detected a total of 339 pathogenic species (Table S1) in five TW sampling sites (Fig. 1A) under stable water (SW) supply in Nanjing with metagenomic sequencing. Eight pathogenic species within four different genera were identified to have high relative abundance (> 0.1% in the bacterial community) in SW samples (Fig. 1B). As the transmission distance increased, the relative abundances in the bacterial community of *Pseudomonas aeruginosa* (0.19–1.61%), *Pseudomonas alcaligenes* (0.24–4.04%), *Sphingomonas paucimobilis* (0.07–0.15%) and *Mycobacterium avium* (0.01–0.27%) increased gradually from JQM to XLW (Fig. 1B). Among all the sampling sites, site JHS showed to have the highest abundance of *Pseudomonas stutzeri* (0.29%), *Pseudomonas fluorescens* (0.20%), *Pseudomonas putida* (0.17%), *Aeromonas hydrophila* (0.04%) and *Salmonella enterica* (0.02%) (Fig. 1B and Fig. S1). However, only the relative abundance of *P. aeruginosa* increased and became the dominant pathogen (Fig. 1B) after re-chlorination tank treatment, from 1.61% in site XLW to 22.55% in site MQ. Moreover, the cluster analysis further illustrated that the structural similarity and alpha diversity of the pathogen community structure in SW decreased with increasing transport distance (Fig. 1B).

Three typical pathogens including *P. aeruginosa*, *M. avium* and *Salmonella* sp. were chosen to detect their absolute abundance due to their prevalence and high relative abundance in SW samples. PMA-qPCR results showed that *P. aeruginosa* had the highest average concentration (7.40 × 10^3^ cells/L) and detection frequency (96%, 48/50 samples), followed by *M. avium* (7.42 × 10^2^ cells/L; 84%, 42/50 samples) and *Salmonella* sp. (27 cells/L; 36%, 18/50 samples). Furthermore, the absolute abundances of all the three pathogens increased generally from site JQM to site XLW and decreased from site XLW to site MQ, but an exception at site RHL for *M. avium* and *Salmonella* sp. (Fig. 1C). In particular, *Salmonella* sp. was significantly affected by the re-chlorination tank between site XLW and site MQ (*p* < 0.001), with a considerable decrease in absolute abundance. One-way ANOVA analysis showed that sampling sites greatly affected the absolute abundance of *Salmonella* sp. (*p* < 0.05).

### Effect of suspension–restoration water supply on potential pathogens in tap water

**Figure 2:**
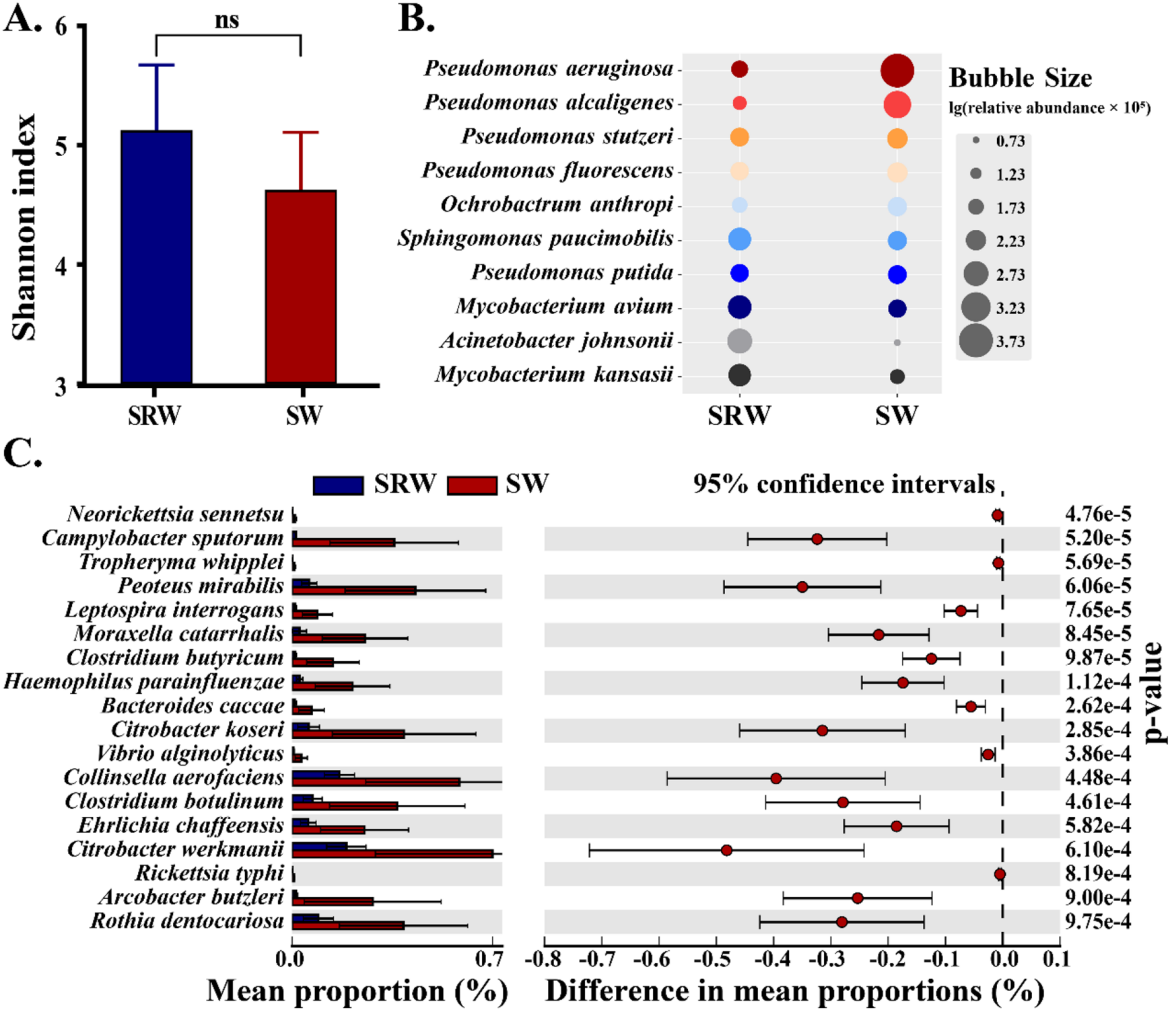
Differences in pathogen community composition under different water supply scenarios. (**A**) Shannon index (mean ± standard error, n = 3 in SRW and n = 15 in SW) of pathogen community showing the difference of alpha diversity between SRW and SW. (**B**) Bubble plot showing the relative abundance of abundant (> 0.1%) pathogens in SW and SRW samples. (**C**) Extended error bar plot showing the pathogens with extremely significant differences (*p* < 0.001) between SRW and SW.

Based on the metagenomic data, we compared differences in the communities of potential pathogens in TW during suspension–restoration water (SRW) supply and stable water supply. Statistical analysis showed that SRW and SW had similar alpha diversity (*p* > 0.05) of the pathogens (Fig. 2A), but some prevalent pathogens had different abundances between the SRW and SW samples. SRW was found to have seven pathogens with high relative abundance of over 0.1%, and SW had eight ones, of which five pathogens were shared with the two water types (Fig. 2B). *P. aeruginosa* (4.91%), *P. alcaligenes* (1.05%) and *Ochrobactrum anthropi* (0.13%) were considered highly abundant in SW exclusively, while *Acinetobacter johnsonii* (0.59%) and *Mycobacterium kansasii* (0.33%) had high abundance in SRW. The predominant pathogen changed from *P. aeruginosa* in SW to *A. johnsonii* in SRW (Fig. 2B). Moreover, SW and SRW were identified to have 78 pathogens with significant difference (*p* < 0.05, Fig. S2) and 18 pathogens with extremely significant difference (*p* < 0.001, Fig. 2C) in the relative abundance. Eight pathogens had higher proportions in SRW group, and 70 ones were highly abundant in SW group (*p* < 0.05, Fig. S2). When *p* < 0.001, all 18 pathogens had higher proportions in SW group (Fig. 2C).

**Figure 3:**
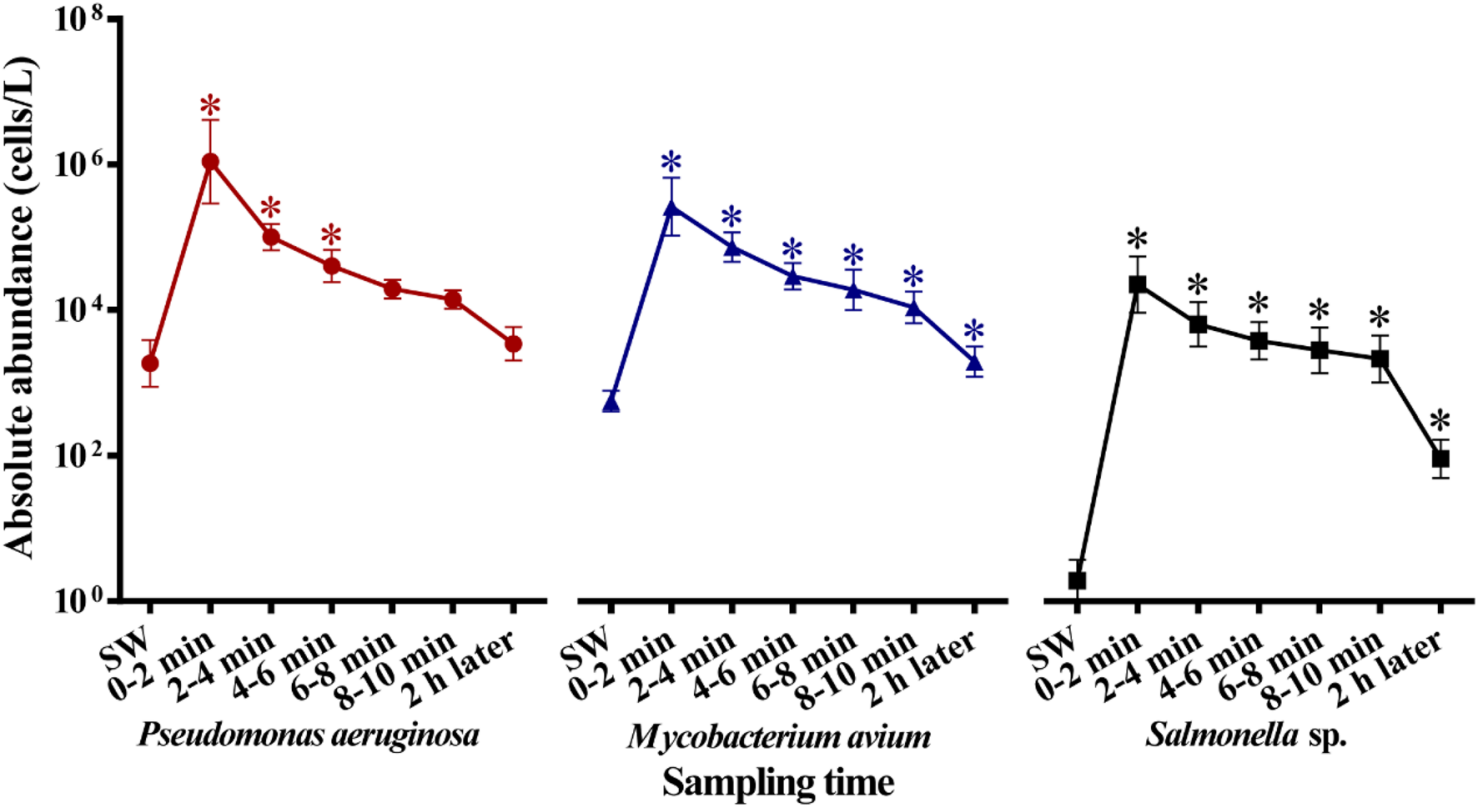
Absolute abundance (mean ± standard error, n = 3) of the three typical pathogens in SRW after water supply restoration. * means significant difference by *t*-test between SRW and SW (*p* < 0.05).

As for absolute quantification, PMA-qPCRs showed that the occurrence frequency of the three pathogens (*P. aeruginosa*, *M. avium* and *Salmonella* sp.) was 100% in SRW, which was substantially higher compared to SW. The largest increase in absolute abundance of *Salmonella* sp. (3.60 log, *p* < 0.05), followed by *P. aeruginosa* (2.58 log, *p* < 0.05) and *M. avium* (2.44 log, *p* < 0.05) was observed within 0–2 min after the restoration of water supply compared to the stable water supply. Moreover, the absolute abundances of the three pathogens in SRW (0–6 min) were significantly higher than those in SW (*p* < 0.05), and decreased with the extension of the water supply restoration time (Fig. 3). The absolute abundance of *P. aeruginosa* in SRW after 6 min of water supply restoration was similar to that in SW (*p* > 0.05). However, the absolute abundances of *M. avium* (1.09 × 10^4^ cells/L) and *Salmonella* sp. (2.14 × 10^3^ cells/L) were significantly higher than those in SW (*p* < 0.05) even after water supply restoration for 2 h.

### Health risk of tap water under different water supply scenarios

**Figure 4:**
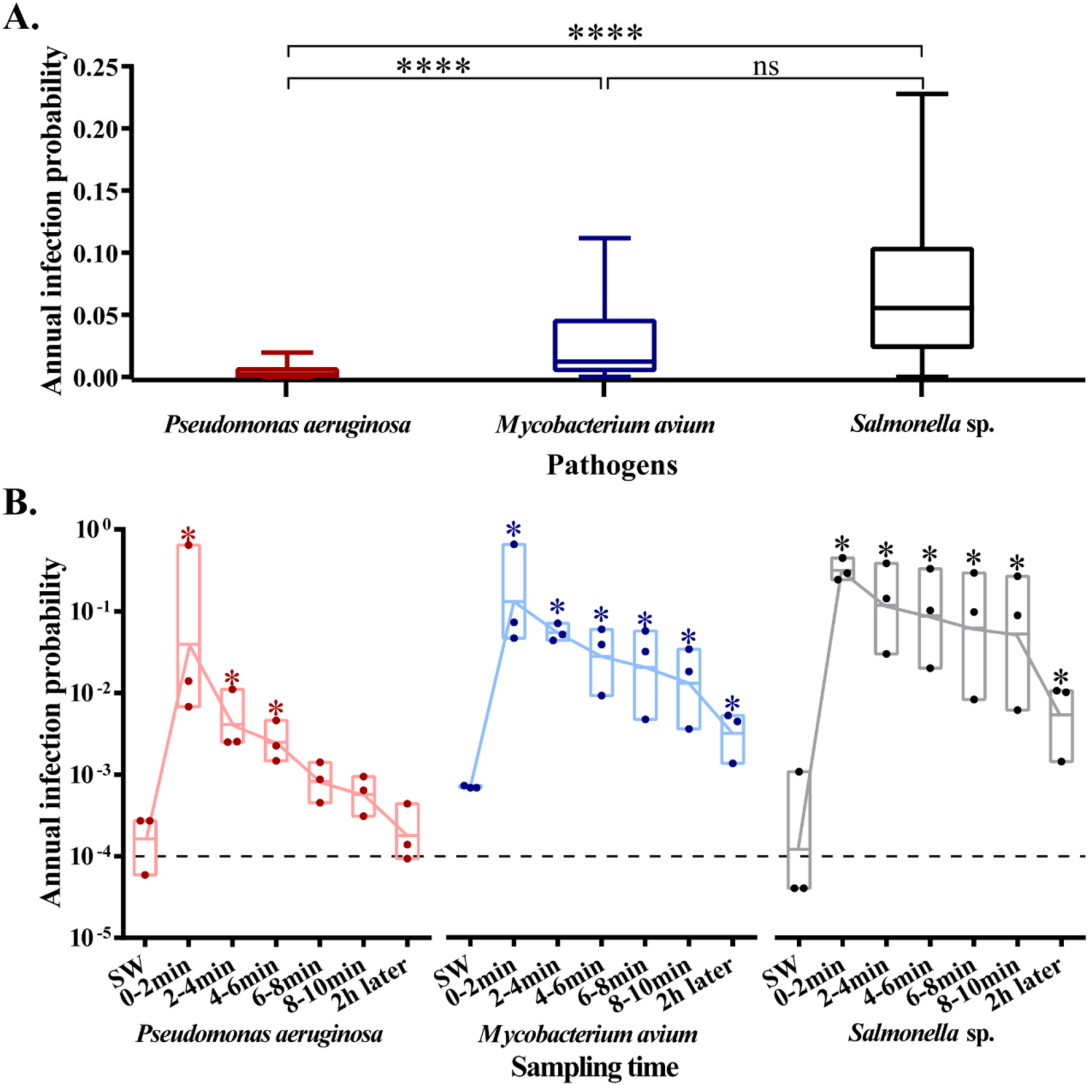
Annual infection probability of the three pathogens in SW (**A**) and SRW (**B**). (**A**) n = 5000. (**B**) n = 3. The exposure routes were dermal exposure of *P. aeruginosa* and oral intake of *M. avium* and *Salmonella* sp.; **** means extremely significant difference and ns means no significant difference by *t*-test between the two pathogens (*p* < 0.0001). * means significant difference by *t*-test between SRW and SW (*p* < 0.05). Dash line means the risk threshold 1 × 10^−4^.

We further calculated the annual infection probabilities P_(inf, y)_ of the three pathogens in each of SW and SRW samples. The absolute abundance data in SW were fitted to probability distribution models with maximum likelihood estimation (Table S2, Fig. S3), which were found to be appropriate by Kolmogorov–Smirnov test (Table S2). Monte Carlo simulation showed that the median P_(inf, y)_ of each of the three pathogens (Fig. 4A) exceeded the risk limit (10^−4^) for TW recommended by US EPA^28^. Furthermore, the median P_(inf, y)_ of *M. avium* (1.24 × 10^−2^) and *Salmonella* sp. (5.54 × 10^−2^) through oral intake were one order of magnitude higher than that of *P. aeruginosa* (1.20 × 10^−3^) through dermal exposure (Fig. 4A). Among the three pathogens, a lower infection risk of *P. aeruginosa* in TW was observed compared to the other two pathogens (*p* < 0.0001, Fig. 4A). However, the difference of P_(inf, y)_ between *M. avium* and *Salmonella* sp. was not significant (*p* > 0.05, Fig. 4A).

The P_(inf, y)_ of the three pathogens in each SRW sample increased significantly (*P. aeruginosa* increased by 3.02 × 10^2^, *M. avium* increased by 1.86 × 10^2^ and *Salmonella* sp. increased by 2.63 × 10^3^, each *p* < 0.05) during 0–2 min after water supply restoration, and showed temporally decreasing trends (Fig. 4B). In comparison with *M. avium* and *Salmonella* sp., *P. aeruginosa* demonstrated a more remarkable decrease of P_(inf, y)_ along with the time extension of water supply restoration. However, the P_(inf, y)_ of each pathogen in SRW was above the threshold within the first 10 min after water supply restoration. Notably, the P_(inf, y)_ of *M. avium* and *Salmonella* sp. remained significantly higher than the threshold in SW (*p* < 0.05) after two hours of the water supply restoration, while the P_(inf, y)_ of *P. aeruginosa* (9.33 × 10^−5^) was lower than that in site SRWA.

**Figure 5:**
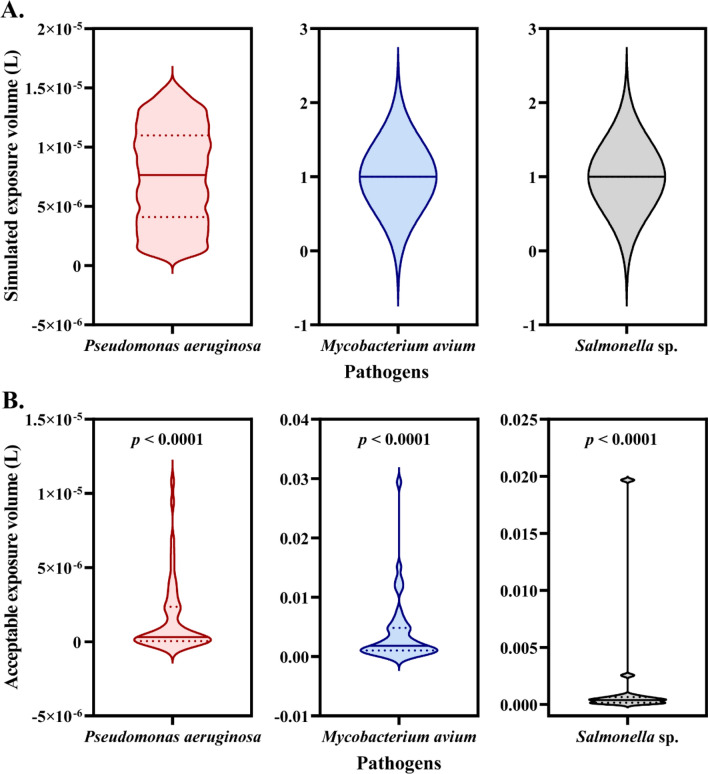
Simulated exposure volume (**A**) and acceptable exposure volume (**B**) of TW for dermal exposure or oral intake. (**A**) n = 5000. (**B**) n = 18. The exposure routes were dermal exposure of *P. aeruginosa* and oral intake of *M. avium* and *Salmonella* sp. *p* < 0.0001 means extremely significant difference by *t*-test between simulated exposure volume and acceptable exposure volume of drinking water.

Moreover, we also calculated the acceptable exposure volume of TW that meets the risk requirement proposed by EPA (10^−4^). The maximum acceptable volume of TW was 1.08 × 10^−5^ L in dermal exposure of *P. aeruginosa*, 2.94 × 10^−2^ L in oral intake of *M. avium* and 1.97 × 10^−2^ L in oral intake of *Salmonella* sp. (Fig. 5B). The acceptable exposure volumes of TW for all three bacteria were significantly lower (*p* < 0.0001, Fig. 5) compared to the exposure volumes adopted in Monte-Carlo simulations, implying high risks arising from direct utilization of TW.

## Discussion

In order to accurately assess the risk for direct utilization of TW, this study gained comprehensive insights into the pathogen communities of TW, and chose key pathogens and optimized the method for absolute quantification. Since PMA pretreatment can eliminate the disturbance of dead bacteria^23^, this study applied PMA-qPCR to determine the cell numbers of bacterial pathogens in the given volume of water to meet the requirements of QMRA^19^. Single-copy genes were chosen to avoid complex conversions and uneven amplification of multi-copy genes, which was often overlooked^29,30^. Bacteria recovery efficiency, DNA extraction efficiency and PCR amplification efficiency were also under consideration to increase the accuracy of absolute quantification of the pathogens. Moreover, based on the infection characteristics of the three pathogens in TW, different exposure routes were also considered in this study to enhance the reliability of QMRA results. *P. aeruginosa* causes eye and skin infections^31,32^, and systemic infections through the injured skin into the patient^33^. Infection caused by *Salmonella* sp. begins with the ingestion of contaminated food or water^34^, allowing pathogens to reach the intestinal epithelium and cause intestinal diseases such as typhoid and paratyphoid fever^35^. *M. avium* can cause zoonotic infection by nasal or oral ingestion and can affect a variety of human tissues and organs, including the lungs, bone marrow and lymph nodes^36^.

In this study, both metagenomic analysis and PMA-qPCR revealed the presence of a variety of bacterial pathogens in TW. *P. aeruginosa* had the highest abundance in TW (especially in site MQ), which indicates that *P. aeruginosa* particularly adapts to the pipeline network environment compared to other pathogens. Both culture-dependent and molecular methods have confirmed the frequent occurrence of *P. aeruginosa* in TW^37,38^. The species is able to tolerate chlorine^39^ due to the superior barrier properties of outer membrane^40^ and remarkable release of extracellular polymeric substances^41^, resulting in its higher abundance than the other species within the genus of *Pseudomonads*^42^. Moreover, chlorination process can induce the over expression of the MexEF-OprN efflux pump^43^ and the promotion of horizontal transfer of plasmids^44^, which may greatly promote antibiotic resistance of *P. aeruginosa* and increased the infection risk.

We found that the abundance and community structure of pathogens varied considerably under different water supply scenarios, which is mainly driven by the bulk water growth and biofilm detachment. Many factors are likely to cause bulk water growth, such as sediment, residual chlorine and TOC. The sediment in the pipe will be stirred up when the water supply is restored after the water supply has been suspended, which may cause an increase in turbidity and the number of bacteria^45^. The concentration of chlorine and TOC was an important factor in limiting the number of bacteria. The residual chlorine concentration decreased and total cell counts increased significantly in TW after stagnation^11^. In addition, a decrease in TOC concentration was observed during water stagnation, which was partly caused by bacteria consumption for growth^46^.

Biofilm provides a possible habitat for pathogens, where microbes are protected against disinfectants^47^. These organisms include fecal indicator bacteria, obligate pathogens of fecal origin, opportunistic pathogens of environmental origin, enteric viruses and parasitic protozoa^48^. In fact, only less than 2% of the bacterial members in a drinking water system are found in the water phase^49^. Thus, bacteria in biofilms enter the pipeline due to the shear stress after water supply suspension–restoration^50,51^, increasing the absolute abundances in SRW of the three typical pathogens (*P. aeruginosa*, *M. avium* and *Salmonella* sp.) that tend to form biofilm in DWDS^52–55^ and can be persistent in biofilm for weeks^56^. Compared to SW, pathogens with increased relative abundance in SRW included *Acinetobacter*, *Sphingomonas* and *Mycobacterium*, the genera with high abundance in pipe biofilm^48,57,58^.

This study revealed that the annual infection risk of the three pathogens in DW were significantly higher than the EPA-designated acceptable threshold (10^−4^) in the case of direct utilization of TW without any treatment. Similarly, some previous studies have showed that the infection probability in many regions exceeded this threshold^59–62^. Besides, the exposure routes selected in this study were direct contact or drinking of TW, but residents in China usually boil TW before consumption. Also, some point-of-use commercial water purifiers like reverse membranes can further decrease the abundance and the health risks of pathogens, which were not taken consideration in this study. Since pathogens are greatly reduced by boiling or membrane treatment^63^, the above results therefore somewhat overestimated the potential human health risk of the pathogens arising from contact or consumption of tap water. Notwithstanding, the finding of this study that health risks of pathogens in SRW is significantly higher than that in SW still makes sense because of the changes in pathogen community and increases of their abundances. In addition, we further compared the simulated exposure volumes used in QMRA with the acceptable exposure volumes when utilized directly, resulting in significantly lower acceptable exposure volumes in this study. These warn us that the risk of TW is higher during a short period after the water supply has been restored. We suggest residents avoid direct consumption of TW if possible, including washing and direct drinking, during this period especially the first two hours.

As for all QMRA approaches, the input variables remain uncertain and limited, including exposure dose, assumptions underlying exposure assessment and dose–response parameters for pathogens used in the model^9^. We compensated for this uncertainty and limitation of exposure dose by several attempts. Considering assumptions underlying exposure assessment, we modeled daily drinking volume of TW as 1 L based on exposure scenarios described in EPA and WHO handbook^5,64^. This probability distribution is hardly representative of water consumption behavior in settings including a complex system of household water management. While for the dose–response parameters in the model, those parameters were collected in high-income setting with healthy adults, which may underestimate the infection risk for populations in developing countries and young children^65^. Drinking and rinsing after heating and differences in consumers were not under consideration due to the lack of relevant parameters. Besides the annual infection probability, many studies have also calculated disability-adjusted life years (DALYs) as the summary measure of disease burden^9,60,66^. However, this component was not taken into consideration in this study by virtue of the absence of relevant epidemiological data.

This study provided a comprehensive insight into the effect of water supply suspension–restoration on human pathogen community composition and their potential human health risks. *P. aeruginosa* was the dominant pathogen in SW samples. Water supply suspension–restoration caused the obvious shift of the community composition of pathogens and the evident increase in the absolute abundances of *P. aeruginosa*, *M. avium* and *Salmonella* sp. The infection risks of the three pathogens arising from direct utilization of drinking water under stable water supply, including dermal exposure and oral intake, were all above the threshold of 10^−4^ recommended by EPA. Water supply suspension–restoration further increased the health risk, demonstrating the necessity that more attempts should be dedicated to controlling the pathogens proliferation induced by water supply suspension.

## Methods

### Water sampling and DNA extraction

Sampling TW under stable water supply (no water cut-off for over one week, SW samples) and suspended–restored water supply (water supply restored after more than six-hours suspension, SRW samples) was carried out to characterize the composition and its variation of bacterial community. The coordinate positions of the sampling sites were shown in Table S3. We collected TW samples with filter elements (MK2-EG-BG, Toray, Japan) for the maximum biomass along the distribution pipeline according to the linear distance from drinking water treatment plant (DWTP) (Fig. 1A) for 10 times (every two weeks from June 14th 2019) and obtained a total of 50 SW samples. Three water supply suspension events (Fig. 1A) were selected by inquiring about the water suspension announcement in advance. Before water supply suspension, 10 L of tap water were collected in SRW site A–C as SW samples. When drinking water supply was restored, we continuously collected tap water (10 L) at five time points within the first ten minutes (0–2 min, 2–4 min, 4–6 min, 6–8 min and 8–10 min), considering that the flow rate was 5.48 L/min. We also collected samples after two hours (2 h later) and the taps were kept running until collection. TW samples in SRW site A–C were filtered with 0.22 μm micropore membrane (BKMAN, China) to collect microorganism, because of limited TW volume. For DNA extraction, the filter elements and micropore membranes were separately soaked with 1 × PBS overnight to elute microorganism and the eluent was centrifuged at 14,000×*g* for 15 min. DNA was extracted using the FastDNA^®^ Spin Kit for Soil (MP Biomedicals, USA), and the DNA concentration and purity were measured by micro-spectrophotometry (NanoDrop One, Thermo Fisher Scientific, China).

### High throughput sequencing and bioinformatics analysis

DNA extracted from the three SW samples (randomly chosen from the 10 time points) collected at each site and the three SRW samples (0–2 min) were subject to metagenome sequencing on the Illumina HiSeq 2500 platform (Novogene Bioinformatic Technology, Beijing, China). The sequencing strategy was Index 150 PE (paired-end sequencing, 150-bp reads) to generate nearly equal number of reads for each sample. The final size of the raw data in FASTQ format was approximately 100 M reads for each sample (Table S4). The raw reads with low quality (more than 10 “N” or 50% bases with Q ≤ 5) or contaminated by adapter were removed by fastp^67^ (version 0.19.7), and the filtered clean reads were used for metagenomic analysis. The sequencing reads of TW samples were annotated by Metaphlan2^68^ with default parameters to obtain species profiles and their relative abundance. In order to identify HPB, the species profiles were compared with the self-established HPB database composed of 534 species (Table S5)^69–71^.

### PMA-qPCR

The single-copy genes, *oaa*, 16S rRNA gene (V1–V2) and *invA* (Table S6) have been identified to be specifically located on the genomes of *P. aeruginosa*^72^, *M. avium*^73^ and *Salmonella* sp.^74,75^, respectively. Thus, the three genes in all the TW samples were quantified by PMA-qPCR to determine their copy numbers. An optimization assay was also designed and conducted to determine the optimal concentration of PMAxx (Biotium, USA), which was finally achieved at 15 μM (Text S1, Fig. S4). qPCRs were conducted with a final volume of 25 μL, containing 12.5 μL of 2 × SYBR Green Mix (Vazyme Biotech, Nanjing, China), 1 μL of each primer (10 μM), 2 μL of template DNA and 8.5 μL of ddH_2_O. Thermal cycling and fluorescence detection were performed under different reaction conditions (Table S7) on QuantStudio 3 Real-Time PCR Systems with QuantStudio Design and Analysis Software (version 1.4) (Thermo Fisher Scientific, China). Each reaction was performed in triplicate. Standard curves (Fig. S5) were obtained with tenfold serial dilutions of the recombinant plasmids carrying target genes^76^, to generate the amplification efficiency and correlation coefficient (R^2^) of the PCRs. Based on the standard curve and the Ct value of each sample, the gene abundance was calculated and normalized against volume (L) of water samples. Stability tests and sensitivity tests were carried out to ensure the feasibility of the optimized PMA-qPCR (details in Text S1, Table S8, Figs. S6 and S7).

### Absolute quantification of pathogens

For absolute quantification of the three pathogens, tap water samples spiked with the three typical pathogens were tested to obtain the recovery efficiencies during the sample pretreatment (details in Text S1). Briefly, the given concentrations of pathogens (Table S9) were added to TW and filtered through filter elements or micropore membrane to collect microorganism. After quantified with the optimized PMA-qPCR method, the concentration of recovered pathogens was correlated to the spiked bacterial concentrations after logarithm transformation to obtain the recovery efficiency for the water filtration and DNA extraction methods (Table S9). Each gradient was set up in triplicate for the experiment.

The number of pathogens per liter (cells/L) in DW, namely the absolute concentration (C), was calculated by the formula:

1 $$C=\frac{Q\cdot {V}_{a}}{{V}_{b}\cdot k}$$

where, *Q* is concentration of extracted DNA eluent quantified with qPCR (copies/μL DNA eluent), *V*_*a*_ is final volume of extracted DNA eluent (μL), *k* is recovery efficiency of targeted bacteria for water filtration and DNA extraction, and *V*_*b*_ is volume of drinking water samples to extract DNA (L).

### Quantitative microbial risk assessment

QMRA of the target pathogens (*P. aeruginosa, Salmonella* sp. and *M. avium*) was conducted by following the most commonly used four-step risk assessment process^77^, including hazard identification, exposure assessment, dose–response assessment and risk characterization (details in Text S2). We chose the three pathogens for QMRA by considering frequency of detection, reliability of detection methods, and availability of dose–response information^77^, based on the data obtained from this study and previous studies (details in Text S2). Two exposure routes, namely oral intake (direct drinking) and dermal exposure (eye contact during washing), were opted for exposure assessment. Beta-Poisson model was used to simulate the dose–response correlations for the three pathogens, and the relevant information has been summarized in Tables S10 and S11. The annual infection probability P_(inf, y)_, the descriptive endpoint of risk assessment, was calculated with the formula shown in Text S2. We ran 5000 simulations using Monte Carlo in Matlab to predict the overall annual infection risk.

### Statistical analyses

Cluster analysis was performed with PAST (version 4.03) for high-abundance pathogens (average relative abundance > 0.1% in all SW samples) identified by metagenomic analysis based on Bray–Curtis distance. Welch’s *t*-test was used to identify pathogens with significant differences (*p* < 0.05) between SRW and SW samples with STAMP^78^ (version 2.1.3). One-way ANOVA and *t*-test were performed using SPSS (IBM, USA) to determine significant difference in absolute abundance and annual infection probability of the pathogens at the different sampling sites, and the results were considered statistically significant when *p* < 0.05.

## Supplementary Information

Supplementary Information.

## Data Availability

All the metagenomic data in this study have been deposited in NCBI Sequence Read Archive under accession number PRJNA807827.
