# Supplementary material for: Shift of human pathogen community composition and their potential human health risk after supply suspension in tap water
Source: Sci Rep. 2023 Aug 1;13:12419. doi: 10.1038/s41598-023-39225-z (PMC10393962; doi:10.1038/s41598-023-39225-z)
Supplement: Supplementary file 1 — Supplementary Information. [file 41598_2023_39225_MOESM1_ESM.pdf]

# Supplementary Information for

# "Shift of Human Pathogen Community Composition and Their Potential Human Health Risk after Supply Suspension in Tap Water"

## Submitted to ***Scientific Reports***

Shengnan Liu [1], Qisheng Li [2], Ruiming Jiang [1], Peng Liu [1]*, Xu-Xiang Zhang [1]*

[1] State Key Laboratory of Pollution Control and Resource Reuse, School of the Environment, Nanjing University, Nanjing 210023, China

[2] China Three Gorges Construction Engineering Corporation, Beijing 100048, China

*** Corresponding author**

Name: Xu-Xiang Zhang; Peng Liu

Addresses: State Key Laboratory of Pollution Control and Resource Reuse, School of the Environment, Nanjing University, 163 Xianlin Road, Nanjing 210023, China.

Phone: +86-25-89680368

Fax: +86-25-89680368

Email: zhangxx@nju.edu.cn (Xu-Xiang Zhang); lp085853@163.com (Peng Liu)

**Total Pages: 48**

**Texts: 2**

**Tables: 11**

**Figures: 7**

## Contents

**Text S1** Optimal PMA-qPCR Experimental Conditions

**Text S2** Quantitative Microbial Risk Assessment

**Table S1** The species of bacterial pathogens present in drinking water detected by aligning metagenomics data against HPB database with Metaphlan2.

**Table S2** Parameters of generalized extreme value and exponentia distributions fitted to the concentrations of the three typical pathogens in drinking water.

**Table S3** The basic information of the metagenomic data obtained by Illumina high-throughput sequencing for the drinking water samples.

**Table S4** The list of human pathogenic bacteria (HPB) in the self-merged database.

**Table S5** Basic information of specific single-copy genes in three typical pathogens.

**Table S6** Basic information of primers and reaction conditions for three typical pathogens.

**Table S7** Comparison between the theoretical number of pathogens and the quantitative result of PMA-qPCR to test sensitivity.

**Table S8** Related information of three pathogens for recovery experiment in drinking water with two filtration methods.

**Table S9** Exposure parameters related to human body washing and rinsing.

**Table S10** Dose-response parameters of the three pathogens.

**Figure S1** Relative abundance of the top 50 pathogens in the different sampling sites.

**Figure S2** Extended error bar plot showing pathogens with significant differences.

**Figure S3** Probability density distribution diagram and Probability Plot of absolute abundance of three pathogens.

**Figure S4** Histogram showing Ct values of live and dead bacteria under PMAxx treatment with different concentrations.

**Figure S5** The standard curve of the three specific single copy genes at the optimal annealing temperature.

**Figure S6** Correlation between Ct value (mean ± standard error) of live bacteria determined by PMA-qPCR and theoretical number of live bacteria.

**Figure S7** Linear relationship between the theoretical live count of three pathogens and the actual quantitative results of PMA-qPCR.

**References**

**Text S1** Optimal PMA-qPCR Experimental Conditions

We optimized the experimental conditions for PMA-qPCR of the three genes *oaa*, 16S rRNA gene (V1 – V2) and *inv*A. Different volumes of PMAxx stock solution were added to 1 mL of suspension of live and autoclaved dead bacteria and mixed thoroughly to achieve final PMAxx concentrations of 0, 5, 10, 15 and 20 μmol/L respectively. The mixture was incubated in the dark for 10 min, and exposed under a PMA-Light with a 650 W halogen tungsten lamp for 15 min. After PMAxx pretreatment, pathogenic DNA was extracted using TIANamp Bacteria DNA Kit (Tiangen Biotech, China) for qPCR of each gene. The optimal concentration of PMAxx pretreatment was obtained by Ct value. Histogram showed that the Ct values of live bacteria did not change significantly ($p > 0.05$) with increasing PMAxx concentration. When the PMAxx concentration was in the range of 0 – 15 μM, the Ct values of dead bacteria increased with PMAxx concentration except for *P. aeruginosa* ($p < 0.05$). Besides, no significant differences ($p > 0.05$) were observed between the Ct values of the dead bacteria treated with 15 and 20 μM PMAxx, which indicated that 15 μM PMAxx was able to completely bind to the DNA of dead pathogens (Figure S4). Hence 15 μM PMAxx was taken for pre-treatment in this study.

Stability tests were carried out by PMA-qPCR with different proportions of live and dead bacteria. Sensitivity tests were carried out by PMA-qPCR with equal volumes of live and dead bacteria mixed in a 10-fold concentration gradient dilution. Three parallel sets of each live and dead bacteria mixture were set up. The optimized PMA-

qPCR was proved stable (Figure S6) and highly sensitive (Figure S7). The sensitivity of PMA-qPCR was high when the theoretical number of *P. aeruginosa*, *M. avium* and *Salmonella* sp. were $4.0\times10^2$ – $4.0\times10^8$ cells, $2.0\times10^2$ – $2.0\times10^8$ cells and $5.0\times10^2$ – $5.0\times10^6$ cells, respectively (Table S8). However, when the theoretical number of the three pathogens were less than 100 cells, the error was too large to obtain a precise and stable quantification of the pathogens.

In addition, we determined the recovery efficiency of DNA. Based on the actual dosing range for the three pathogens (Table S9), the three bacterial solutions were diluted in a 5-fold gradient for a total of 5 gradients in triplicate. The diluted bacterial solutions were added to 2 L and 10 L of drinking water, mixed and then filtered through filter elements and micropore membranes for biomass, respectively. The filter elements and micropore membranes were eluted with 1 × PBS, treated with the optimized PMA-qPCR and the numbers of the three pathogens were calculated. At the same time, the recovery efficiency standard curves (the logarithm of the actual number of pathogens recovered versus the logarithm of the theoretical number of pathogens injected) were plotted to obtain the recovery efficiency of the three pathogens under two filtration methods separately. DNA recovery efficiency experiment results showed a high correlation ($R^2 > 0.99$) between the actual recovery dose and the actual added dose of pathogens (Table S9). Moreover, the recovery efficiency of 0.22 μm micropore membrane was higher than that of filter element (Table S8).

**Text S2** Quantitative Microbial Risk Assessment

We followed the most commonly used four-step method [1,2] for quantitative microbial risk assessment, namely hazard identification, exposure assessment, dose-response assessment and risk characterization.

**Hazard identification**. Tap water was closely linked to the health of urban residents. The selection of these reference pathogens (*P. aeruginosa*, *Salmonella* sp. and *M. avium*) was based on several criteria including commonly detected, reliable detection methods, harmful to human health and available dose-response information [1], which was considered reasonable in previous studies and this study. *P. aeruginosa* and *M. avium* were opportunistic premise plumbing pathogens [3]. *P. aeruginosa* could infect the human eyes and skins [4]. *M. avium* instigated lung infections [5] and was significant to morbidity and mortality of acquired immune deficiency syndrome (AIDS) [6]. Infection by *Salmonella* sp. caused intestinal diseases [7] such as typhoid and paratyphoid fever, which killed an estimated 135,900 people worldwide in 2017 [8].

**Exposure assessment.** PMA-qPCRs were performed to obtain the concentration of pathogens in DW samples in this study. Exposure dose (d) was equal to the concentration of pathogens in DW (C) times volume of intake or exposure to DW (V). The concentration of pathogens in DW (C) was calculated according to absolute quantitative results of PMA-qPCR. The intake/exposure volume of DW was divided into two exposure routes to calculate according to the different pathogenic mechanisms: oral intake (direct drinking, the adult daily volume of water one liter) of *Salmonella* sp.

and *M. avium* and dermal exposure (eye contact during washing) of *P. aeruginosa*. Table S9 shows the formula and related parameters of dermal exposure.

**Dose-response assessment.** The dose-response model for the three pathogens was Beta-Poisson, common in QMRA, which was used to model the probability of the three pathogens surviving and causing disease in humans. The dose-response information of the three pathogens were summarized in Tables S9 and S10.

**Risk characterization.** The annual infection probability $P_{(inf,y)}$ was regarded as the descriptive endpoint of risk characterization. The $P_{(inf,y)}$ of the three pathogens in DW was calculated through formula (2) – (3). However, the $P_{(inf,y)}$ in SRW was calculated based on the frequency of five occurrences per year, since the city has lower frequency of water supply suspension. In addition, the probability distribution of absolute abundance of pathogens in SW was fitted by the maximum likelihood method and Kolmogorov-Smirnov test was performed for goodness of fit. Monte Carlo simulation was applied to predict the $P_{(inf,y)}$ by selecting values randomly from the optimum model 5000 times. All the $P_{(inf,y)}$ were used to fit cumulative probability in order to observe probability of annual infection risk caused by three pathogens below the risk threshold $1\times10^{-4}$ [9].

$$P_{(inf,d)} = 1 - [1 + \frac{d}{N_{50}} \cdot (2^{\frac{1}{\alpha}} - 1)]^{-\alpha} \quad (2)$$

$$P_{(inf,y)} = 1 - (1 - P_{(inf,d)})^{365} \quad (3)$$

where, $P_{(inf,d)}$ is the daily infection probability, $d$ is the exposure dose , $\alpha$ and $\beta$ are the dose-response parameters for the different pathogens, $N_{50}$ is the half infectious dose,

and $P_{(inf, y)}$ is the annual infection probability.

**Table S1** The species of bacterial pathogens present in drinking water detected by aligning metagenomics data against HPB database with Metaphlan2.

| No. | Pathogen |
|---|---|
| 1 | *Abiotrophia defectiva* |
| 2 | *Acidaminococcus fermentans* |
| 3 | *Acinetobacter haemolyticus* |
| 4 | *Acinetobacter lwoffii* |
| 5 | *Actinobacillus hominis* |
| 6 | *Actinobacillus suis* |
| 7 | *Actinomyces gerencseriae* |
| 8 | *Actinomyces naeslundii* |
| 9 | *Actinomyces radingae* |
| 10 | *Aeromonas caviae* |
| 11 | *Aeromonas veronii* |
| 12 | *Aggregatibacter segnis* |
| 13 | *Anaerococcus lactolyticus* |
| 14 | *Anaplasma phagocytophilum* |
| 15 | *Arcanobacterium pyogenes* |
| 16 | *Bacillus anthracis* |
| 17 | *Bacillus coagulans* |
| 18 | *Bacillus pumilus* |
| 19 | *Bacillus thuringiensis* |
| 20 | *Bacteroides eggerthii* |
| 21 | *Bacteroides galacturonicus* |
| 22 | *Bacteroides ovatus* |
| 23 | *Bacteroides stercoris* |
| 24 | *Bacteroides ureolyticus* |
| 25 | *Bartonella bacilliformis* |
| 26 | *Bartonella quintana* |
| 27 | *Bilophila wadsworthia* |
| 28 | *Bordetella pertussis* |
| 29 | *Borrelia duttonii* |
| 30 | *Borrelia latyschewii* |
| 31 | *Borrelia recurrentis* |
| 32 | *Brevundimonas diminuta* |
| 33 | *Brucella melitensis* |
| 34 | *Burkholderia mallei* |
| 35 | *Campylobacter concisus* |
| 36 | *Campylobacter gracilis* |
| 37 | *Campylobacter lari* |
| 38 | *Campylobacter upsaliensis* |

| No. | Pathogen |
|---|---|
| 39 | *Capnocytophaga gingivalis* |
| 40 | *Cardiobacterium hominis* |
| 41 | *Cedecea neteri* |
| 42 | *Chlamydia pneumoniae* |
| 43 | *Chlamydophila pneumoniae* |
| 44 | *Chryseobacterium meningosepticum* |
| 45 | *Citrobacter farmeri* |
| 46 | *Citrobacter rodentium* |
| 47 | *Citrobacter youngae* |
| 48 | *Clostridium bifermentans* |
| 49 | *Clostridium chauvoei* |
| 50 | *Clostridium histolyticum* |
| 51 | *Clostridium ramosum* |
| 52 | *Clostridium sporogenes* |
| 53 | *Collinsella aerofaciens* |
| 54 | *Corynebacterium amycolatum* |
| 55 | *Corynebacterium diphtheriae* |
| 56 | *Corynebacterium macginleyi* |
| 57 | *Corynebacterium pseudodiphthericum* |
| 58 | *Corynebacterium striatum* |
| 59 | *Corynebacterium xerosis* |
| 60 | *Delftia acidovorans* |
| 61 | *Edwardsiella hoshinae* |
| 62 | *Ehrlichia chaffeensis* |
| 63 | *Enterobacter aerogenes* |
| 64 | *Enterobacter cancerogenus* |
| 65 | *Enterobacter hormaechei* |
| 66 | *Enterococcus casseliflavus* |
| 67 | *Enterococcus faecium* |
| 68 | *Enterococcus mundtii* |
| 69 | *Escherichia coli* |
| 70 | *Eubacterium contortum* |
| 71 | *Eubacterium moniliforme* |
| 72 | *Eubacterium rectale* |
| 73 | *Eubacterium sulci* |
| 74 | *Fibrobacter intestinalis* |
| 75 | *Fluoribacter bozemanae* |
| 76 | *Francisella cf.* |
| 77 | *Fusobacterium necrophorum* |
| 78 | *Fusobacterium ulcerans* |
| 79 | *Gemella morbillorum* |

| No. | Pathogen |
|---|---|
| 80 | *Gordonia rubripertincta* |
| 81 | *Granulicatella adiacens* |
| 82 | *Haemophilus haemolyticus* |
| 83 | *Haemophilus parainfluenzae* |
| 84 | *Hafnia alvei* |
| 85 | *Helicobacter heilmannii* |
| 86 | *Kingella denitrificans* |
| 87 | *Klebsiella oxytoca* |
| 88 | *Kluyvera cryocrescens* |
| 89 | *Legionella birminghamensis* |
| 90 | *Legionella feeleii* |
| 91 | *Legionella lansingensis* |
| 92 | *Legionella oakridgensis* |
| 93 | *Legionella sainthelensi* |
| 94 | *Leifsonia aquatica* |
| 95 | *Leptospira interrogans* |
| 96 | *Leptospira noguchii* |
| 97 | *Leptotrichia buccalis* |
| 98 | *Listeria seeligeri* |
| 99 | *Megamonas hypermegale* |
| 100 | *Moraxella atlantae* |
| 101 | *Moraxella caviae* |
| 102 | *Moraxella lincolnii* |
| 103 | *Moraxella ovis* |
| 104 | *Mycobacterium africanum* |
| 105 | *Mycobacterium bovis* |
| 106 | *Mycobacterium conspicuum* |
| 107 | *Mycobacterium gordonae* |
| 108 | *Mycobacterium kansasii* |
| 109 | *Mycobacterium marinum* |
| 110 | *Mycobacterium porcinum* |
| 111 | *Mycobacterium shimoidei* |
| 112 | *Mycobacterium szulgai* |
| 113 | *Mycobacterium xenopi* |
| 114 | *Mycoplasma hominis* |
| 115 | *Myroides odoratus* |
| 116 | *Neisseria flava* |
| 117 | *Neisseria lactamica* |
| 118 | *Neisseria perflava* |
| 119 | *Neisseria weaveri* |
| 120 | *Nocardia brasiliensis* |

| No. | Pathogen |
|---|---|
| 121 | *Nocardia otitidiscaviarum* |
| 122 | *Ochrobactrum anthropi* |
| 123 | *Oligella urethralis* |
| 124 | *Paenibacillus macerans* |
| 125 | *Pasteurella caballi* |
| 126 | *Pasteurella multocida* |
| 127 | *Peptococcus niger* |
| 128 | *Photobacterium damselae* |
| 129 | *Porphyromonas catoniae* |
| 130 | *Porphyromonas gingivalis* |
| 131 | *Prevotella bivia* |
| 132 | *Prevotella corporis* |
| 133 | *Prevotella disiens* |
| 134 | *Prevotella loescheii* |
| 135 | *Prevotella oralis* |
| 136 | *Prevotella ruminicola* |
| 137 | *Propionibacterium acnes* |
| 138 | *Propionibacterium propionicum* |
| 139 | *Proteus vulgaris* |
| 140 | *Providencia stuartii* |
| 141 | *Pseudomonas fluorescens* |
| 142 | *Pseudomonas stutzeri* |
| 143 | *Psychrobacter phenylpyruvicus* |
| 144 | *Rhodococcus equi* |
| 145 | *Rhodococcus rhodnii* |
| 146 | *Rickettsia akari* |
| 147 | *Rickettsia felis* |
| 148 | *Rickettsia massiliae* |
| 149 | *Rickettsia sibirica* |
| 150 | *Saccharomonospora viridis* |
| 151 | *Salmonella enterica* |
| 152 | *Salmonella typhimurium* |
| 153 | *Selenomonas dianae* |
| 154 | *Selenomonas noxia* |
| 155 | *Serratia odorifera* |
| 156 | *Serratia rubidaea* |
| 157 | *Shigella flexneri* |
| 158 | *Staphylococcus aureus* |
| 159 | *Staphylococcus hyicus* |
| 160 | *Staphylococcus saprophyticus* |
| 161 | *Streptobacillus moniliformis* |

| No. | Pathogen |
|---|---|
| 162 | *Streptococcus anginosus* |
| 163 | *Streptococcus constellatus* |
| 164 | *Streptococcus milleri* |
| 165 | *Streptococcus pyogenes* |
| 166 | *Streptococcus sobrinus* |
| 167 | *Sutterella wadsworthensis* |
| 168 | *Tatlockia micdadei* |
| 169 | *Tropheryma whippelii* |
| 170 | *Tsukamurella paurometabola* |
| 171 | *Ureaplasma urealyticum* |
| 172 | *Veillonella parvula* |
| 173 | *Vibrio cincinnatiensis* |
| 174 | *Vibrio mimicus* |
| 175 | *Welshimeri gene* |
| 176 | *Yersinia enterocolitica* |
| 177 | *Yersinia kristensenii* |
| 178 | *Yersinia pseudotuberculosis* |
| 179 | *Achromobacter piechaudii* |
| 180 | *Acinetobacter baumannii* |
| 181 | *Acinetobacter johnsonii* |
| 182 | *Acinetobacter radioresistens* |
| 183 | *Actinobacillus lignieresii* |
| 184 | *Actinobacillus ureae* |
| 185 | *Actinomyces israelii* |
| 186 | *Actinomyces neuii* |
| 187 | *Actinomyces turicensis* |
| 188 | *Aeromonas hydrophila* |
| 189 | *Aggregatibacter actinomycetemcomitans* |
| 190 | *Alcaligenes faecalis* |
| 191 | *Anaerococcus prevotii* |
| 192 | *Arcanobacterium bernardiae* |
| 193 | *Arcobacter butzleri* |
| 194 | *Bacillus cereus* |
| 195 | *Bacillus licheniformis* |
| 196 | *Bacillus sphaericus* |
| 197 | *Bacteroides caccae* |
| 198 | *Bacteroides forsythus* |
| 199 | *Bacteroides heparinolyticus* |
| 200 | *Bacteroides pectinophilus* |
| 201 | *Bacteroides thetaiotaomicron* |
| 202 | *Bacteroides vulgatus* |

| No. | Pathogen |
|---|---|
| 203 | *Bartonella elizabethae* |
| 204 | *Bergeyella zoohelcum* |
| 205 | *Bordetella avium* |
| 206 | *Borrelia burgdorferi* |
| 207 | *Borrelia hermsii* |
| 208 | *Borrelia parkeri* |
| 209 | *Borrelia turicatae* |
| 210 | *Brevundimonas vesicularis* |
| 211 | *Brucella suis* |
| 212 | *Burkholderia pseudomallei* |
| 213 | *Campylobacter curvus* |
| 214 | *Campylobacter hyointestinalis* |
| 215 | *Campylobacter rectus,* |
| 216 | *Capnocytophaga canimorsus* |
| 217 | *Capnocytophaga ochracea* |
| 218 | *Cedecea davisae* |
| 219 | *Cellulomonas cellulans* |
| 220 | *Chlamydia psittaci* |
| 221 | *Chromobacterium violaceum* |
| 222 | *Citrobacter amalonaticus* |
| 223 | *Citrobacter freundii* |
| 224 | *Citrobacter sedlakii* |
| 225 | *Clostridioides difficile* |
| 226 | *Clostridium botulinum* |
| 227 | *Clostridium difficile* |
| 228 | *Clostridium novyi* |
| 229 | *Clostridium septicum* |
| 230 | *Clostridium tertium* |
| 231 | *Comamonas testosteroni* |
| 232 | *Corynebacterium argentoratense* |
| 233 | *Corynebacterium jeikeium* |
| 234 | *Corynebacterium minutissimum* |
| 235 | *Corynebacterium pseudodiphtheriticum* |
| 236 | *Corynebacterium ulcerans* |
| 237 | *Coxiella burnetii* |
| 238 | *Dermatophilus congolensis* |
| 239 | *Edwardsiella tarda* |
| 240 | *Ehrlichia ewingii* |
| 241 | *Enterobacter amnigenus* |
| 242 | *Enterobacter cloacae* |
| 243 | *Enterobacter sakazakii* |

| No. | Pathogen |
|---|---|
| 244 | *Enterococcus durans* |
| 245 | *Enterococcus gallinarum* |
| 246 | *Enterococcus raffinosus* |
| 247 | *Eubacterium brachy* |
| 248 | *Eubacterium cylindroides* |
| 249 | *Eubacterium multiforme* |
| 250 | *Eubacterium saburreum* |
| 251 | *Eubacterium tenue* |
| 252 | *Filifactor alocis* |
| 253 | *Fluoribacter dumoffii* |
| 254 | *Francisella tularensis* |
| 255 | *Fusobacterium nucleatum* |
| 256 | *Fusobacterium varium* |
| 257 | *Gordonia amarae* |
| 258 | *Gordonia sputi* |
| 259 | *Grimontia hollisae* |
| 260 | *Haemophilus influenzae* |
| 261 | *Haemophilus paraphrophilus* |
| 262 | *Helicobacter cinaedi* |
| 263 | *Helicobacter pullorum* |
| 264 | *Kingella kingae* |
| 265 | *Klebsiella pneumoniae* |
| 266 | *Lactobacillus sp.* |
| 267 | *Legionella cherrii* |
| 268 | *Legionella hackeliae* |
| 269 | *Legionella longbeachae* |
| 270 | *Legionella pneumophila* |
| 271 | *Legionella tucsonensis* |
| 272 | *Leptospira borgpetersenii* |
| 273 | *Leptospira kirschneri* |
| 274 | *Leptospira santarosai* |
| 275 | *Listeria ivanovii* |
| 276 | *Listeria welshimeri* |
| 277 | *Micromonas micros* |
| 278 | *Moraxella bovis* |
| 279 | *Moraxella cuniculi* |
| 280 | *Moraxella nonliquefaciens* |
| 281 | *Morganella morganii* |
| 282 | *Mycobacterium asiaticum* |
| 283 | *Mycobacterium celatum* |
| 284 | *Mycobacterium fortuitum* |

| No. | Pathogen |
|---|---|
| 285 | *Mycobacterium haemophilum* |
| 286 | *Mycobacterium leprae* |
| 287 | *Mycobacterium mucogenicum* |
| 288 | *Mycobacterium scrofulaceum* |
| 289 | *Mycobacterium simiae* |
| 290 | *Mycobacterium tuberculosis* |
| 291 | *Mycoplasma fermentans* |
| 292 | *Mycoplasma pneumoniae* |
| 293 | *Neisseria cinerea* |
| 294 | *Neisseria flavescens* |
| 295 | *Neisseria meningitidis* |
| 296 | *Neisseria sicca* |
| 297 | *Neorickettsia sennetsu* |
| 298 | *Nocardia farcinica* |
| 299 | *Nocardia pseudobrasiliensis* |
| 300 | *Odoribacter splanchnicus* |
| 301 | *Orientia tsutsugamushi* |
| 302 | *Pantoea agglomerans* |
| 303 | *Pasteurella canis* |
| 304 | *Pasteurella pneumotropica* |
| 305 | *Peptoniphilus asaccharolyticus* |
| 306 | *Plesiomonas shigelloides* |
| 307 | *Porphyromonas circumdentaria* |
| 308 | *Porphyromonas levii* |
| 309 | *Prevotella buccae* |
| 310 | *Prevotella dentalis* |
| 311 | *Prevotella enoeca* |
| 312 | *Prevotella melaninogenica* |
| 313 | *Prevotella oris* |
| 314 | *Prevotella tannerae* |
| 315 | *Propionibacterium avidum* |
| 316 | *Proteus mirabilis* |
| 317 | *Providencia alcalifaciens* |
| 318 | *Pseudomonas aeruginosa* |
| 319 | *Pseudomonas pseudoalcaligenes* |
| 320 | *Pseudonocardia autotrophica* |
| 321 | *Rahnella aquatilis* |
| 322 | *Rhodococcus erythropolis* |
| 323 | *Rhodococcus rhodochrous* |
| 324 | *Rickettsia australis* |
| 325 | *Rickettsia honei* |

| No. | Pathogen |
|---|---|
| 326 | *Rickettsia prowazekii* |
| 327 | *Rickettsia typhi* |
| 328 | *Saccharopolyspora rectivirgula* |
| 329 | *Salmonella enteritidis* |
| 330 | *Sebaldella termitidis* |
| 331 | *Selenomonas flueggei* |
| 332 | *Serratia ficaria* |
| 333 | *Serratia plymuthica* |
| 334 | *Shigella boydii* |
| 335 | *Shigella sonnei* |
| 336 | *Staphylococcus epidermidis* |
| 337 | *Staphylococcus intermedius* |
| 338 | *Staphylococcus warneri* |
| 339 | *Streptococcus acidominimus* |
| 340 | *Streptococcus bovis* |
| 341 | *Streptococcus criceti* |
| 342 | *Streptococcus mitis* |
| 343 | *Streptococcus salivarius* |
| 344 | *Streptococcus suis* |
| 345 | *Suttonella indologenes* |
| 346 | *Tatumella ptyseos* |
| 347 | *Tropheryma whipplei* |
| 348 | *Tsukamurella pulmonis* |
| 349 | *Veillonella atypica* |
| 350 | *Vibrio alginolyticus* |
| 351 | *Vibrio fluvialis* |
| 352 | *Vibrio parahaemolyticus* |
| 353 | *Wolinella succinogenes* |
| 354 | *Yersinia frederiksenii* |
| 355 | *Yersinia mollaretii* |
| 356 | *Yersinia rohdei* |
| 357 | *Achromobacter xylosoxidans* |
| 358 | *Acinetobacter calcoaceticus* |
| 359 | *Acinetobacter junii* |
| 360 | *Actinobacillus equuli* |
| 361 | *Actinobacillus pleuropneumoniae* |
| 362 | *Actinomyces georgiae* |
| 363 | *Actinomyces meyeri* |
| 364 | *Actinomyces odontolyticus* |
| 365 | *Aerococcus viridans* |
| 366 | *Aeromonas sobria* |

| No. | Pathogen |
|---|---|
| 367 | *Aggregatibacter aphrophilus* |
| 368 | *Amycolatopsis orientalis* |
| 369 | *Anaerococcus vaginalis* |
| 370 | *Arcanobacterium haemolyticum* |
| 371 | *Arcobacter cryaerophilus* |
| 372 | *Bacillus circulans* |
| 373 | *Bacillus mycoides* |
| 374 | *Bacillus subtilis* |
| 375 | *Bacteroides distasonis* |
| 376 | *Bacteroides fragilis* |
| 377 | *Bacteroides merdae* |
| 378 | *Bacteroides splanchnicus* |
| 379 | *Bacteroides uniformis* |
| 380 | *Bacteroides zoogleoformans* |
| 381 | *Bartonella henselae* |
| 382 | *Bifidobacterium dentium* |
| 383 | *Bordetella bronchiseptica* |
| 384 | *Borrelia crocidurae* |
| 385 | *Borrelia hispanica* |
| 386 | *Borrelia persica* |
| 387 | *Brevibacillus brevis* |
| 388 | *Brucella abortus* |
| 389 | *Burkholderia cepacia* |
| 390 | *Campylobacter coli* |
| 391 | *Campylobacter fetus* |
| 392 | *Campylobacter jejuni* |
| 393 | *Campylobacter sputorum* |
| 394 | *Capnocytophaga cynodegmi* |
| 395 | *Capnocytophaga sputigena* |
| 396 | *Cedecea lapagei* |
| 397 | *Centipeda periodontii* |
| 398 | *Chlamydia trachomatis* |
| 399 | *Chryseobacterium balustinum* |
| 400 | *Citrobacter braakii* |
| 401 | *Citrobacter koseri* |
| 402 | *Citrobacter werkmanii* |
| 403 | *Clostridium baratii* |
| 404 | *Clostridium butyricum* |
| 405 | *Clostridium fallax* |
| 406 | *Clostridium perfringens* |
| 407 | *Clostridium sordellii* |

| No. | Pathogen |
|---|---|
| 408 | *Clostridium tetani* |
| 409 | *Corynebacterium afermentans* |
| 410 | *Corynebacterium bovis* |
| 411 | *Corynebacterium kutscheri* |
| 412 | *Corynebacterium propinquum* |
| 413 | *Corynebacterium pseudotuberculosis* |
| 414 | *Corynebacterium urealyticum* |
| 415 | *Cronobacter sakazakii* |
| 416 | *Dichelobacter nodosus* |
| 417 | *Eggerthella lenta* |
| 418 | *Eikenella corrodens* |
| 419 | *Enterobacter asburiae* |
| 420 | *Enterobacter gergoviae* |
| 421 | *Enterococcus avium* |
| 422 | *Enterococcus faecalis* |
| 423 | *Enterococcus hirae* |
| 424 | *Erysipelothrix rhusiopathiae* |
| 425 | *Eubacterium combesii* |
| 426 | *Eubacterium limosum* |
| 427 | *Eubacterium nodatum* |
| 428 | *Eubacterium saphenum* |
| 429 | *Ewingella americana* |
| 430 | *Finegoldia magna* |
| 431 | *Fluoribacter gormanii* |
| 432 | *Fusobacterium mortiferum* |
| 433 | *Fusobacterium periodonticum* |
| 434 | *Gardnerella vaginalis* |
| 435 | *Gordonia bronchialis* |
| 436 | *Gordonia terrae* |
| 437 | *Haemophilus ducreyi* |
| 438 | *Haemophilus parahaemolyticus* |
| 439 | *Haemophilus segnis* |
| 440 | *Helicobacter fennelliae* |
| 441 | *Helicobacter pylori* |
| 442 | *Klebsiella granulomatis* |
| 443 | *Kluyvera ascorbata* |
| 444 | *Legionella anisa* |
| 445 | *Legionella cincinnatiensis* |
| 446 | *Legionella jordanis* |
| 447 | *Legionella maceachernii* |
| 448 | *Legionella rubrilucens* |

| No. | Pathogen |
|---|---|
| 449 | *Legionella wadsworthii* |
| 450 | *Leptospira inadai* |
| 451 | *Leptospira meyeri* |
| 452 | *Leptospira weilii* |
| 453 | *Listeria monocytogenes* |
| 454 | *Mannheimia haemolytica* |
| 455 | *Mogibacterium timidum* |
| 456 | *Moraxella catarrhalis* |
| 457 | *Moraxella lacunata* |
| 458 | *Moraxella osloensis* |
| 459 | *Mycobacterium abscessus* |
| 460 | *Mycobacterium avium* |
| 461 | *Mycobacterium chelonae* |
| 462 | *Mycobacterium genavense* |
| 463 | *Mycobacterium kansaii* |
| 464 | *Mycobacterium malmoense* |
| 465 | *Mycobacterium peregrinum* |
| 466 | *Mycobacterium senegalense* |
| 467 | *Mycobacterium smegmatis* |
| 468 | *Mycobacterium ulcerans* |
| 469 | *Mycoplasma genitalium* |
| 470 | *Mycoplasma salivarium* |
| 471 | *Neisseria elongata* |
| 472 | *Neisseria gonorrhoeae* |
| 473 | *Neisseria mucosa* |
| 474 | *Neisseria subflava* |
| 475 | *Nocardia asteroides* |
| 476 | *Nocardia nova* |
| 477 | *Nocardia transvalensis* |
| 478 | *Oligella ureolytica* |
| 479 | *Paenibacillus alvei* |
| 480 | *Pasteurella aerogenes* |
| 481 | *Pasteurella dagmatis* |
| 482 | *Pasteurella stomatis* |
| 483 | *Peptostreptococcus anaerobius* |
| 484 | *Porphyromonas asaccharolytica* |
| 485 | *Porphyromonas endodontalis* |
| 486 | *Porphyromonas salivosa* |
| 487 | *Prevotella buccalis* |
| 488 | *Prevotella denticola* |
| 489 | *Prevotella intermedia* |

| No. | Pathogen |
|---|---|
| 490 | *Prevotella nigrescens* |
| 491 | *Prevotella oulora* |
| 492 | *Prevotella veroralis* |
| 493 | *Propionibacterium granulosum* |
| 494 | *Proteus penneri* |
| 495 | *Providencia rettgeri* |
| 496 | *Pseudomonas alcaligenes* |
| 497 | *Pseudomonas putida* |
| 498 | *Pseudoramibacter alactolyticus* |
| 499 | *Ralstonia pickettii* |
| 500 | *Rhodococcus fascians* |
| 501 | *Rickettsia africae* |
| 502 | *Rickettsia conorii* |
| 503 | *Rickettsia japonica* |
| 504 | *Rickettsia rickettsii* |
| 505 | *Rothia dentocariosa* |
| 506 | *Salmonella bongori* |
| 507 | *Salmonella typhi* |
| 508 | *Selenomonas artemidis* |
| 509 | *Selenomonas infelix* |
| 510 | *Serratia marcescens* |
| 511 | *Serratia proteamaculans* |
| 512 | *Shigella dysenteriae* |
| 513 | *Sphingomonas paucimobilis* |
| 514 | *Staphylococcus haemolyticus* |
| 515 | *Staphylococcus lugdunensis* |
| 516 | *Stenotrophomonas maltophilia* |
| 517 | *Streptococcus agalactiae* |
| 518 | *Streptococcus canis* |
| 519 | *Streptococcus equi* |
| 520 | *Streptococcus pneumoniae* |
| 521 | *Streptococcus sanguis* |
| 522 | *Streptococcus uberis* |
| 523 | *Tannerella forsythia* |
| 524 | *Treponema pallidum* |
| 525 | *Tsukamurella inchonensis* |
| 526 | *Tsukamurella tyrosinosolvens* |
| 527 | *Veillonella dispar* |
| 528 | *Vibrio cholerae* |
| 529 | *Vibrio furnissii* |
| 530 | *Vibrio vulnificus* |

| No. | Pathogen |
|---|---|
| 531 | *Yersinia bercovieri* |
| 532 | *Yersinia intermedia* |
| 533 | *Yersinia pestis* |
| 534 | *Yersinia ruckeri* |

**Table S2** Parameters of generalized extreme value and exponentia distributions fitted to the concentrations of the three typical pathogens in drinking water.

| Pathogens | Type of probability distribution | Formula | Parameters | K-S test |
|---|---|---|---|---|
| *Pseudomonas aeruginosa* | Generalized Extreme Value | $f(x)=(\frac{1}{\sigma})exp\left\{-\left[1+k\frac{(x-\mu)}{\sigma}\right]^{-\frac{1}{k}}\right\}\left[1+k\frac{(x-\mu)}{\sigma}\right]^{-1-\frac{1}{k}}$ | k=2.03598<br>σ=713.68<br>μ=335.195 | h=0 |
| *Mycobacterium avium* | Generalized Extreme Value | $f(x)=(\frac{1}{\sigma})exp\left\{-\left[1+k\frac{(x-\mu)}{\sigma}\right]^{-\frac{1}{k}}\right\}\left[1+k\frac{(x-\mu)}{\sigma}\right]^{-1-\frac{1}{k}}$ | k=1.38116<br>σ=179.394<br>μ=102.174 | h=0 |
| *Salmonella* sp. | Exponentia | $f(x)=\frac{1}{\mu}e^{-\frac{x}{\mu}}$ | μ=26.5556 | h=0 |

**Note:** "h=0": fails to reject the null hypothesis at the default 5% significance level.

Table S3 The coordinate positions of the sampling points.

| Sampling points | X | Y |
| --- | --- | --- |
| DWTP | 118.727 | 32.048 |
| JQM | 118.746 | 32.034 |
| RHL | 118.749 | 32.096 |
| JHS | 118.812 | 32.063 |
| XLW | 118.865 | 32.041 |
| re-chlorination tank | 118.883 | 32.050 |
| MQ | 118.901 | 32.056 |
| SRWA | 118.803 | 32.056 |
| SRWB | 118.745 | 32.089 |
| SRWC | 118.777 | 32.099 |

**Table S4** The basic information of the metagenomic data obtained by Illumina high-throughput sequencing for the drinking water samples.

| Group | Samples | | Raw dataset read number | Clean dataset read number | Average length (bp) |
|---|---|---|---|---|---|
| SW | SWA | SWA-1 | 101,171,576 | 78,272,606 | 150 |
| | | SWA-2 | 155,431,704 | 113,155,548 | |
| | | SWA-3 | 125,811,946 | 94,276,786 | |
| | SWB | SWB-1 | 139,342,542 | 105,313,258 | 150 |
| | | SWB-2 | 162,181,000 | 115,208,302 | |
| | | SWB-3 | 118,469,724 | 85,040,372 | |
| | SWC | SWC-1 | 129,907,022 | 97,139,158 | 150 |
| | | SWC-2 | 139,771,836 | 97,896,112 | |
| | | SWC-3 | 152,920,454 | 109,333,226 | |
| | SWD | SWD-1 | 147,971,212 | 113,462,084 | 150 |
| | | SWD-2 | 141,381,482 | 102,530,102 | |
| | | SWD-3 | 149,678,990 | 109,687,986 | |
| | SWE | SWE1 | 136,324,968 | 105,586,392 | 150 |
| | | SWE2 | 102,011,758 | 76,462,818 | |
| | | SWE3 | 173,351,294 | 123,026,668 | |
| SRW | SRW-1 | | 80,126,000 | 58,454,618 | 150 |
| | SRW-2 | | 86,307,586 | 62,973,354 | |
| | SRW-3 | | 75,817,256 | 53,319,338 | |

**Table S5** The list of human pathogenic bacteria (HPB) in the self-merged database.

| No. | Pathogen |
|---|---|
| 1 | *Achromobacter xylosoxidans* |
| 2 | *Acidaminococcus fermentans* |
| 3 | *Acinetobacter baumannii* |
| 4 | *Acinetobacter calcoaceticus* |
| 5 | *Acinetobacter haemolyticus* |
| 6 | *Acinetobacter johnsonii* |
| 7 | *Acinetobacter junii* |
| 8 | *Acinetobacter lwoffii* |
| 9 | *Acinetobacter radioresistens* |
| 10 | *Actinobacillus equuli* |
| 11 | *Actinobacillus lignieresii* |
| 12 | *Actinobacillus pleuropneumoniae* |
| 13 | *Actinobacillus suis* |
| 14 | *Actinomyces israelii* |
| 15 | *Actinomyces naeslundii* |
| 16 | *Aerococcus viridans* |
| 17 | *Aeromonas caviae* |
| 18 | *Aeromonas hydrophila* |
| 19 | *Aeromonas veronii* |
| 20 | *Aggregatibacter actinomycetemcomitans* |
| 21 | *Aggregatibacter aphrophilus* |
| 22 | *Aggregatibacter segnis* |
| 23 | *Alcaligenes faecalis* |
| 24 | *Amycolatopsis orientalis* |
| 25 | *Anaerococcus prevotii* |
| 26 | *Anaplasma phagocytophilum* |
| 27 | *Arcanobacterium haemolyticum* |
| 28 | *Arcobacter butzleri* |
| 29 | *Arcobacter cryaerophilus* |
| 30 | *Bacillus anthracis* |
| 31 | *Bacillus cereus* |
| 32 | *Bacillus circulans* |
| 33 | *Bacillus coagulans* |
| 34 | *Bacillus licheniformis* |
| 35 | *Bacillus mycoides* |
| 36 | *Bacillus pumilus* |
| 37 | *Bacillus subtilis* |
| 38 | *Bacillus thuringiensis* |
| 39 | *Bacteroides caccae* |
| 40 | *Bacteroides fragilis* |
| 41 | *Bacteroides heparinolyticus* |

| No. | Pathogen |
|---|---|
| 42 | *Bacteroides ovatus* |
| 43 | *Bacteroides thetaiotaomicron* |
| 44 | *Bacteroides uniformis* |
| 45 | *Bacteroides vulgatus* |
| 46 | *Bacteroides zoogleoformans* |
| 47 | *Bartonella bacilliformis* |
| 48 | *Bartonella elizabethae* |
| 49 | *Bartonella henselae* |
| 50 | *Bartonella quintana* |
| 51 | *Bifidobacterium dentium* |
| 52 | *Bordetella avium* |
| 53 | *Bordetella bronchiseptica* |
| 54 | *Bordetella pertussis* |
| 55 | *Borrelia crocidurae* |
| 56 | *Borrelia duttonii* |
| 57 | *Borrelia hermsii* |
| 58 | *Borrelia parkeri* |
| 59 | *Borrelia recurrentis* |
| 60 | *Borrelia turicatae* |
| 61 | *Brevibacillus brevis* |
| 62 | *Brevundimonas diminuta* |
| 63 | *Brevundimonas vesicularis* |
| 64 | *Brucella abortus* |
| 65 | *Brucella melitensis* |
| 66 | *Brucella suis* |
| 67 | *Burkholderia cepacia* |
| 68 | *Burkholderia mallei* |
| 69 | *Burkholderia pseudomallei* |
| 70 | *Campylobacter coli* |
| 71 | *Campylobacter concisus* |
| 72 | *Campylobacter curvus* |
| 73 | *Campylobacter fetus* |
| 74 | *Campylobacter gracilis* |
| 75 | *Campylobacter hyointestinalis* |
| 76 | *Campylobacter jejuni* |
| 77 | *Campylobacter lari* |
| 78 | *Campylobacter sputorum* |
| 79 | *Campylobacter upsaliensis* |
| 80 | *Capnocytophaga canimorsus* |
| 81 | *Capnocytophaga cynodegmi* |
| 82 | *Capnocytophaga gingivalis* |
| 83 | *Capnocytophaga ochracea* |
| 84 | *Capnocytophaga sputigena* |

| No. | Pathogen |
|---|---|
| 85 | *Cardiobacterium hominis* |
| 86 | *Cedecea lapagei* |
| 87 | *Cedecea neteri* |
| 88 | *Chlamydia pneumoniae* |
| 89 | *Chlamydia psittaci* |
| 90 | *Chlamydia trachomatis* |
| 91 | *Chromobacterium violaceum* |
| 92 | *Chryseobacterium balustinum* |
| 93 | *Citrobacter amalonaticus* |
| 94 | *Citrobacter braakii* |
| 95 | *Citrobacter farmeri* |
| 96 | *Citrobacter freundii* |
| 97 | *Citrobacter koseri* |
| 98 | *Citrobacter rodentium* |
| 99 | *Citrobacter werkmanii* |
| 100 | *Citrobacter youngae* |
| 101 | *Clostridioides difficile* |
| 102 | *Clostridium baratii* |
| 103 | *Clostridium botulinum* |
| 104 | *Clostridium butyricum* |
| 105 | *Clostridium chauvoei* |
| 106 | *Clostridium novyi* |
| 107 | *Clostridium perfringens* |
| 108 | *Clostridium septicum* |
| 109 | *Clostridium sporogenes* |
| 110 | *Clostridium tetani* |
| 111 | *Collinsella aerofaciens* |
| 112 | *Comamonas testosteroni* |
| 113 | *Corynebacterium argentoratense* |
| 114 | *Corynebacterium diphtheriae* |
| 115 | *Corynebacterium jeikeium* |
| 116 | *Corynebacterium kutscheri* |
| 117 | *Corynebacterium pseudotuberculosis* |
| 118 | *Corynebacterium striatum* |
| 119 | *Corynebacterium ulcerans* |
| 120 | *Corynebacterium urealyticum* |
| 121 | *Corynebacterium xerosis* |
| 122 | *Coxiella burnetii* |
| 123 | *Cronobacter sakazakii* |
| 124 | *Delftia acidovorans* |
| 125 | *Dermatophilus congolensis* |
| 126 | *Dichelobacter nodosus* |
| 127 | *Edwardsiella hoshinae* |

| No. | Pathogen |
|---|---|
| 128 | *Edwardsiella tarda* |
| 129 | *Eggerthella lenta* |
| 130 | *Ehrlichia chaffeensis* |
| 131 | *Eikenella corrodens* |
| 132 | *Enterobacter asburiae* |
| 133 | *Enterobacter cancerogenus* |
| 134 | *Enterobacter cloacae* |
| 135 | *Enterobacter hormaechei* |
| 136 | *Enterococcus avium* |
| 137 | *Enterococcus casseliflavus* |
| 138 | *Enterococcus durans* |
| 139 | *Enterococcus faecalis* |
| 140 | *Enterococcus faecium* |
| 141 | *Enterococcus gallinarum* |
| 142 | *Enterococcus hirae* |
| 143 | *Enterococcus mundtii* |
| 144 | *Erysipelothrix rhusiopathiae* |
| 145 | *Escherichia coli* |
| 146 | *Eubacterium limosum* |
| 147 | *Filifactor alocis* |
| 148 | *Finegoldia magna* |
| 149 | *Fluoribacter dumoffii* |
| 150 | *Francisella tularensis* |
| 151 | *Fusobacterium mortiferum* |
| 152 | *Fusobacterium necrophorum* |
| 153 | *Fusobacterium nucleatum* |
| 154 | *Fusobacterium periodonticum* |
| 155 | *Fusobacterium ulcerans* |
| 156 | *Fusobacterium varium* |
| 157 | *Gardnerella vaginalis* |
| 158 | *Gemella morbillorum* |
| 159 | *Gordonia bronchialis* |
| 160 | *Gordonia rubripertincta* |
| 161 | *Gordonia terrae* |
| 162 | *Grimontia hollisae* |
| 163 | *Haemophilus haemolyticus* |
| 164 | *Haemophilus influenzae* |
| 165 | *Haemophilus parahaemolyticus* |
| 166 | *Haemophilus parainfluenzae* |
| 167 | *Hafnia alvei* |
| 168 | *Helicobacter cinaedi* |
| 169 | *Helicobacter heilmannii* |
| 170 | *Helicobacter pullorum* |

| No. | Pathogen |
|---|---|
| 171 | *Helicobacter pylori* |
| 172 | *Kingella kingae* |
| 173 | *Klebsiella oxytoca* |
| 174 | *Klebsiella pneumoniae* |
| 175 | *Legionella anisa* |
| 176 | *Legionella cherrii* |
| 177 | *Legionella hackeliae* |
| 178 | *Legionella jordanis* |
| 179 | *Legionella lansingensis* |
| 180 | *Legionella longbeachae* |
| 181 | *Legionella oakridgensis* |
| 182 | *Legionella pneumophila* |
| 183 | *Legionella sainthelensi* |
| 184 | *Leptospira borgpetersenii* |
| 185 | *Leptospira interrogans* |
| 186 | *Leptospira santarosai* |
| 187 | *Leptospira weilii* |
| 188 | *Leptotrichia buccalis* |
| 189 | *Listeria ivanovii* |
| 190 | *Listeria monocytogenes* |
| 191 | *Listeria seeligeri* |
| 192 | *Listeria welshimeri* |
| 193 | *Mannheimia haemolytica* |
| 194 | *Megamonas hypermegale* |
| 195 | *Moraxella bovis* |
| 196 | *Moraxella catarrhalis* |
| 197 | *Moraxella cuniculi* |
| 198 | *Moraxella osloensis* |
| 199 | *Moraxella ovis* |
| 200 | *Morganella morganii* |
| 201 | *Mycobacterium avium* |
| 202 | *Mycobacterium haemophilum* |
| 203 | *Mycobacterium kansasii* |
| 204 | *Mycobacterium leprae* |
| 205 | *Mycobacterium marinum* |
| 206 | *Mycobacterium tuberculosis* |
| 207 | *Mycobacterium ulcerans* |
| 208 | *Mycoplasma fermentans* |
| 209 | *Mycoplasma genitalium* |
| 210 | *Mycoplasma hominis* |
| 211 | *Mycoplasma pneumoniae* |
| 212 | *Mycoplasma salivarium* |
| 213 | *Myroides odoratus* |

| No. | Pathogen |
| --- | --- |
| 214 | *Neisseria cinerea* |
| 215 | *Neisseria elongata* |
| 216 | *Neisseria flavescens* |
| 217 | *Neisseria gonorrhoeae* |
| 218 | *Neisseria lactamica* |
| 219 | *Neisseria meningitidis* |
| 220 | *Neisseria mucosa* |
| 221 | *Neisseria subflava* |
| 222 | *Neisseria weaveri* |
| 223 | *Neorickettsia sennetsu* |
| 224 | *Nocardia asteroides* |
| 225 | *Nocardia brasiliensis* |
| 226 | *Nocardia farcinica* |
| 227 | *Nocardia nova* |
| 228 | *Nocardia otitidiscaviarum* |
| 229 | *Ochrobactrum anthropi* |
| 230 | *Odoribacter splanchnicus* |
| 231 | *Oligella urethralis* |
| 232 | *Orientia tsutsugamushi* |
| 233 | *Paenibacillus alvei* |
| 234 | *Pantoea agglomerans* |
| 235 | *Pasteurella dagmatis* |
| 236 | *Pasteurella multocida* |
| 237 | *Photobacterium damselae* |
| 238 | *Plesiomonas shigelloides* |
| 239 | *Porphyromonas asaccharolytica* |
| 240 | *Porphyromonas gingivalis* |
| 241 | *Prevotella dentalis* |
| 242 | *Prevotella denticola* |
| 243 | *Prevotella enoeca* |
| 244 | *Prevotella intermedia* |
| 245 | *Prevotella melaninogenica* |
| 246 | *Prevotella oris* |
| 247 | *Prevotella ruminicola* |
| 248 | *Proteus mirabilis* |
| 249 | *Proteus vulgaris* |
| 250 | *Providencia alcalifaciens* |
| 251 | *Providencia rettgeri* |
| 252 | *Providencia stuartii* |
| 253 | *Pseudomonas aeruginosa* |
| 254 | *Pseudomonas alcaligenes* |
| 255 | *Pseudomonas fluorescens* |
| 256 | *Pseudomonas putida* |

| No. | Pathogen |
|---|---|
| 257 | *Pseudomonas stutzeri* |
| 258 | *Pseudonocardia autotrophica* |
| 259 | *Rahnella aquatilis* |
| 260 | *Ralstonia pickettii* |
| 261 | *Rhodococcus erythropolis* |
| 262 | *Rhodococcus fascians* |
| 263 | *Rhodococcus rhodochrous* |
| 264 | *Rickettsia africae* |
| 265 | *Rickettsia akari* |
| 266 | *Rickettsia australis* |
| 267 | *Rickettsia conorii* |
| 268 | *Rickettsia felis* |
| 269 | *Rickettsia japonica* |
| 270 | *Rickettsia massiliae* |
| 271 | *Rickettsia prowazekii* |
| 272 | *Rickettsia rickettsii* |
| 273 | *Rickettsia sibirica* |
| 274 | *Rickettsia typhi* |
| 275 | *Rothia dentocariosa* |
| 276 | *Saccharomonospora viridis* |
| 277 | *Salmonella bongori* |
| 278 | *Salmonella enterica* |
| 279 | *Sebaldella termitidis* |
| 280 | *Serratia ficaria* |
| 281 | *Serratia marcescens* |
| 282 | *Serratia odorifera* |
| 283 | *Serratia plymuthica* |
| 284 | *Serratia proteamaculans* |
| 285 | *Serratia rubidaea* |
| 286 | *Shigella boydii* |
| 287 | *Shigella dysenteriae* |
| 288 | *Shigella flexneri* |
| 289 | *Shigella sonnei* |
| 290 | *Sphingomonas paucimobilis* |
| 291 | *Staphylococcus aureus* |
| 292 | *Staphylococcus epidermidis* |
| 293 | *Staphylococcus haemolyticus* |
| 294 | *Staphylococcus hyicus* |
| 295 | *Staphylococcus lugdunensis* |
| 296 | *Staphylococcus saprophyticus* |
| 297 | *Staphylococcus warneri* |
| 298 | *Stenotrophomonas maltophilia* |
| 299 | *Streptobacillus moniliformis* |

| No. | Pathogen |
|---|---|
| 300 | *Streptococcus acidominimus* |
| 301 | *Streptococcus agalactiae* |
| 302 | *Streptococcus anginosus* |
| 303 | *Streptococcus canis* |
| 304 | *Streptococcus constellatus* |
| 305 | *Streptococcus equi* |
| 306 | *Streptococcus milleri* |
| 307 | *Streptococcus mitis* |
| 308 | *Streptococcus pneumoniae* |
| 309 | *Streptococcus pyogenes* |
| 310 | *Streptococcus salivarius* |
| 311 | *Streptococcus sobrinus* |
| 312 | *Streptococcus suis* |
| 313 | *Streptococcus uberis* |
| 314 | *Tannerella forsythia* |
| 315 | *Tatlockia micdadei* |
| 316 | *Tatumella ptyseos* |
| 317 | *Treponema pallidum* |
| 318 | *Tropheryma whipplei* |
| 319 | *Tsukamurella paurometabola* |
| 320 | *Tsukamurella tyrosinosolvens* |
| 321 | *Ureaplasma urealyticum* |
| 322 | *Veillonella dispar* |
| 323 | *Veillonella parvula* |
| 324 | *Vibrio alginolyticus* |
| 325 | *Vibrio cholerae* |
| 326 | *Vibrio fluvialis* |
| 327 | *Vibrio furnissii* |
| 328 | *Vibrio mimicus* |
| 329 | *Vibrio parahaemolyticus* |
| 330 | *Vibrio vulnificus* |
| 331 | *Wolinella succinogenes* |
| 332 | *Yersinia enterocolitica* |
| 333 | *Yersinia frederiksenii* |
| 334 | *Yersinia intermedia* |
| 335 | *Yersinia kristensenii* |
| 336 | *Yersinia pestis* |
| 337 | *Yersinia pseudotuberculosis* |
| 338 | *Yersinia rohdei* |
| 339 | *Yersinia ruckeri* |

**Table S6** Basic information of specific single-copy genes in the three typical pathogens.

| Pathogens | Genes | NCBI number | Gene length (bp) | Gene sequence |
|---|---|---|---|---|
| *Pseudomonas aeruginosa* | *O-antigen acetylase* gene (*oaa*) | CP039990.1 | 232 | ctgggtcgaaaggtggttgttatccatgtacagcgaccagccatc<br>acgttcggcgaaacaatccaggccatcgcagagcacgctggag<br>gggtcgagcacgctcacccgcgggttgccttcggccaacgcatc<br>gaacagcgcattcacgtaggcgacgcgcgcagcgtgctcagcg<br>aagggaaaacgtgcgcgcccgacatcctccccgctcagactca |
| *Mycobacterium avium* | 16S rRNA gene (V1 – V2) | AP020326.1 | 193 | agagtttgatcctggctcaggacgaacgctggcggcgtgcttaac<br>acatgcaagtcgaacggaaaggcctcttcggaggtactcgagtg<br>gcgaacgggtgagtaacacgtgggcaatctgccctgcacttcgg<br>gataagcctgggaaactgggtctaataccggataggacctcaag<br>acgcatgtcttctggt |
| *Salmonella* sp. | *invA* | CP033387.1 | 285 | gtgaaattatcgccacgttcgggcaattcgttattggcgatagcct<br>ggcggtgggttttgttgtcttctctattgtcaccgtggtccagtttatc<br>gttattaccaaaggttcagaacgcgtcgcggaagttgcggcccg<br>attttctctggatggtatgcccggtaaacagatgagtattgatgccg<br>atttgaaggccggtattattgatgcggatgccgcgcgcgaacggc<br>gaagcgtactggaaagggaaagccagctttacggttcctttgacg |

**Table S7** Basic information of primers and reaction conditions for the three typical pathogens.

| Pathogens | Genes | Primer | Primer sequence (5'-3') | Reaction conditions |
|---|---|---|---|---|
| *Pseudomonas aeruginosa* | *oaa* | PA431CF | ctgggtcgaaaggtggttgttatc | 40 cycles: 95°C, 10 s; 56°C, 30 s; 72°C, 30 s |
| | | PA431CR | gcggctggtgcggctgagtc | |
| *Mycobacterium avium* | 16S rRNA gene (V1-V2) | myavF | agagtttgatcctggctcag | 40 cycles: 95°C, 10 s; 55°C, 30 s; 72°C, 30 s |
| | | myavR | accagaagacatgcgtcttg | |
| *Salmonella* sp. | *invA* | 139 | gtgaaattatcgccacgttcgggcaa | 40 cycles: 95°C, 10 s; 52°C, 30 s; 72°C, 30 s |
| | | 141 | tcatcgcaccgtcaaaggaacc | |

**Table S8** Comparison between the theoretical number of pathogens and the quantitative result of PMA-qPCR to test sensitivity.

| Pathogens | Theoretical number (cells) | Result of PMA-qPCR (cells) | Absolute value of the error (%) |
|---|---|---|---|
| *Pseudomonas aeruginosa* | $4.0\times10^{8}$ | $3.96\times10^{8}\pm8.33\times10^{6}$ | 1.08 – 3.08 |
| | $4.0\times10^{7}$ | $4.00\times10^{7}\pm1.57\times10^{6}$ | 3.92 – 3.92 |
| | $4.0\times10^{6}$ | $3.89\times10^{6}\pm9.32\times10^{4}$ | 0.42 – 5.08 |
| | $4.0\times10^{5}$ | $4.03\times10^{5}\pm9.79\times10^{3}$ | 1.70 – 3.20 |
| | $4.0\times10^{4}$ | $3.83\times10^{4}\pm1.09\times10^{3}$ | 1.53 – 6.98 |
| | $4.0\times10^{3}$ | $4.12\times10^{3}\pm1.16\times10^{2}$ | 1.00 – 5.90 |
| | $4.0\times10^{2}$ | $3.79\times10^{2}\pm1.41\times10^{1}$ | 1.73 – 8.76 |
| | $4.0\times10^{1}$ | $1.74\times10^{2}\pm3.23\times10^{1}$ | 254.25 – 415.75 |
| | $4.0\times10^{0}$ | - | - |
| *Mycobacterium avium* | $2.0\times10^{8}$ | $2.01\times10^{8}\pm9.53\times10^{6}$ | 4.27 – 5.27 |
| | $2.0\times10^{7}$ | $2.12\times10^{7}\pm5.11\times10^{5}$ | 3.45 – 8.56 |
| | $2.0\times10^{6}$ | $1.99\times10^{6}\pm1.64\times10^{5}$ | 7.70 – 8.70 |
| | $2.0\times10^{5}$ | $1.89\times10^{5}\pm7.74\times10^{3}$ | 1.63 – 9.37 |
| | $2.0\times10^{4}$ | $1.98\times10^{4}\pm1.19\times10^{3}$ | 4.95 – 6.95 |

| Pathogens | Theoretical number (cells) | Result of PMA-qPCR (cells) | Absolute value of the error (%) |
|---|---|---|---|
| | $2.0\times10^{3}$ | $1.88\times10^{3}\pm4.07\times10^{1}$ | 3.97 – 8.04 |
| | $2.0\times10^{2}$ | $1.91\times10^{2}\pm1.01\times10^{1}$ | 0.55 – 9.55 |
| | $2.0\times10^{1}$ | $1.54\times10^{2}\pm1.11\times10^{1}$ | 614.50 – 725.50 |
| | $2.0\times10^{0}$ | - | - |
| *Salmonella* sp. | $5.0\times10^{6}$ | $4.89\times10^{6}\pm7.02\times10^{4}$ | 0.80 – 3.60 |
| | $5.0\times10^{5}$ | $5.03\times10^{5}\pm7.91\times10^{3}$ | 0.98 – 2.18 |
| | $5.0\times10^{4}$ | $4.93\times10^{4}\pm1.49\times10^{3}$ | 1.58 – 4.38 |
| | $5.0\times10^{3}$ | $4.84\times10^{3}\pm1.76\times10^{2}$ | 0.32 – 6.72 |
| | $5.0\times10^{2}$ | $4.98\times10^{2}\pm2.07\times10^{1}$ | 3.74 – 4.54 |
| | $5.0\times10^{1}$ | $3.74\times10^{2}\pm4.44\times10^{1}$ | 559.20 – 736.80 |
| | $5.0\times10^{0}$ | - | - |

**Note:** "-": below the detection limit of qPCR.

**Table S9** Related information of the three pathogens for recovery experiment in drinking water with two filtration methods.

| Filtration method | Related parameter | *Pseudomonas aeruginosa* | *Mycobacterium avium* | *Salmonella* sp. |
|---|---|---|---|---|
| Filter element | Added dose (cells) | $10^{7.09} – 10^{8.30}$ | $10^{4.60} – 10^{7.30}$ | $10^{4.27} – 10^{5.47}$ |
| | Recovery dose (cells) | $10^{5.90±0.08} – 10^{7.32±0.03}$ | $10^{4.53±0.10} – 10^{6.45±0.01}$ | $10^{2.85±0.08} – 10^{4.70±0.02}$ |
| | Correlation analysis formula | $y = 1.192x - 2.580$ $R^2 = 0.9987$ | $y = 0.6996x + 1.328$ $R^2 = 0.9925$ | $y = 1.546x - 3.721$ $R^2 = 0.9956$ |
| | Recovery efficiency (%) | 10.30 | 13.59 | 17.67 |
| 0.22 μm micropore membrane | Added dose (cells) | $10^{4.98} – 10^{7.77}$ | $10^{3.85} – 10^{6.65}$ | $10^{2.98} – 10^{5.84}$ |
| | Recovery dose (cells) | $10^{3.64±0.10} – 10^{6.33±0.03}$ | $10^{4.57±0.10} – 10^{7.55±0.03}$ | $10^{2.89±0.11} – 10^{5.63±0.04}$ |
| | Correlation analysis formula | $y = 1.044x - 0.5061$ $R^2 = 0.9926$ | $y = 0.9393x + 0.1063$ $R^2 = 0.9933$ | $y = 0.9660x - 0.1102$ $R^2 = 0.9912$ |
| | Recovery efficiency (%) | 59.05 | 47.25 | 61.39 |

**Note:** "y" and "x" means actual recovery and added dose of pathogens.

**Table S10** Exposure parameters related to human body washing and rinsing [10].

| Exposure parameters | Data or formula | Probability distribution |
|---|---|---|
| Hand-eye contact area ($A/mm^2$) | min=100/3 $mm^2$<br>max=2000/3 $mm^2$ | Uniform distribution |
| Thickness of water film in contact (h/mm) | min=$1.97\times10^{-2}$ mm<br>max=$2.34\times10^{-2}$ mm | Uniform distribution |
| Frequency of washing and rinsing (f) | Twice per day | - |
| Exposure volume (V/L) | $V = A \cdot h \cdot f \cdot 10^{-6}$ | - |

**Note:** "-": no related parameters.

**Table S11** Dose-response parameters of the three pathogens.

| Pathogens | α | $N_{50}$ | Reference |
|---|---|---|---|
| *Pseudomonas aeruginosa* | 0.190 | $1.85\times10^{4}$ | [11] |
| *Mycobacterium avium* | 0.201 | $3.51\times10^{7}$ | [12] |
| *Salmonella* sp. | 0.175 | $1.11\times10^{6}$ | [13] |

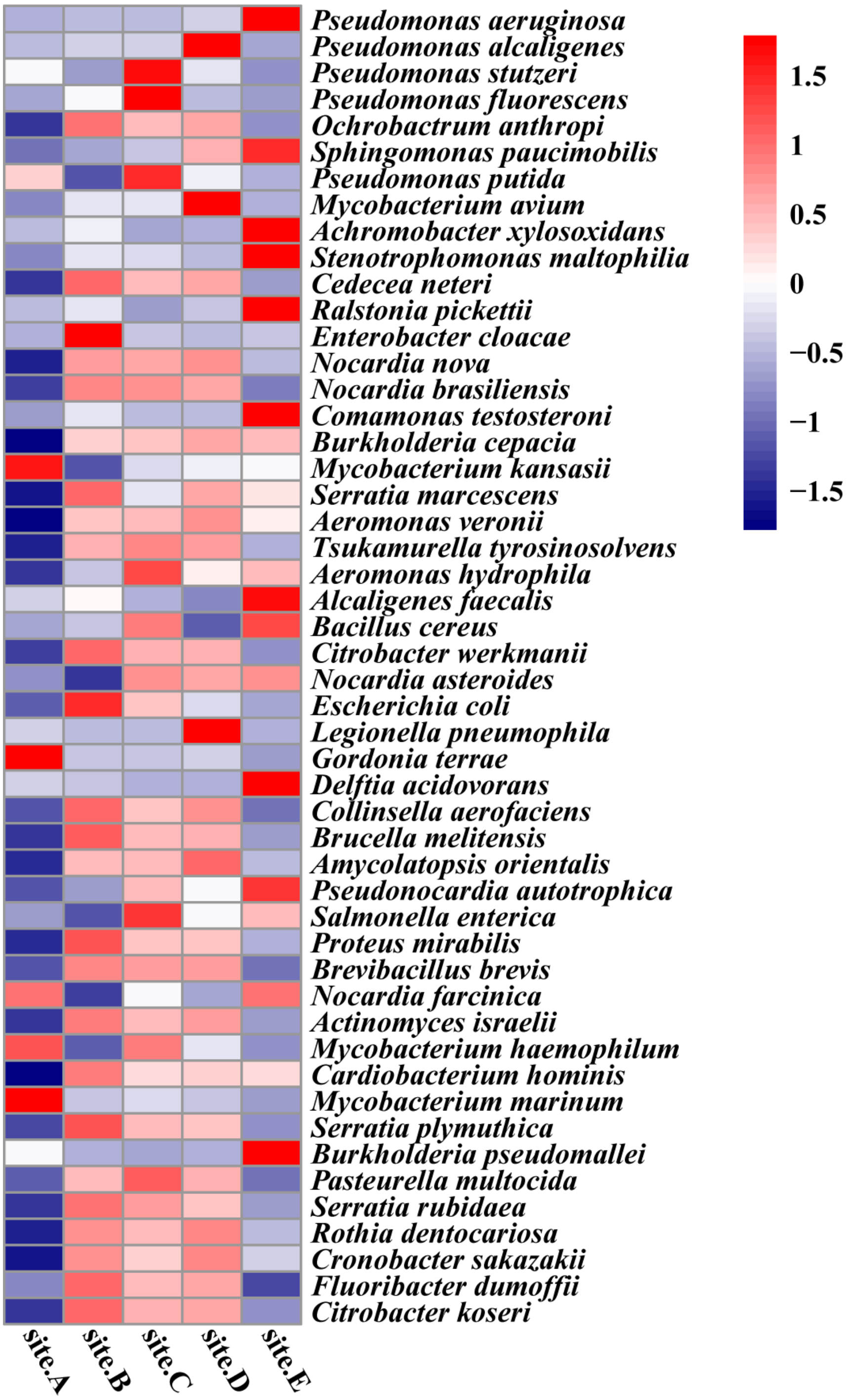


**Figure S1** Relative abundance of the top 50 pathogens in different sampling sites.

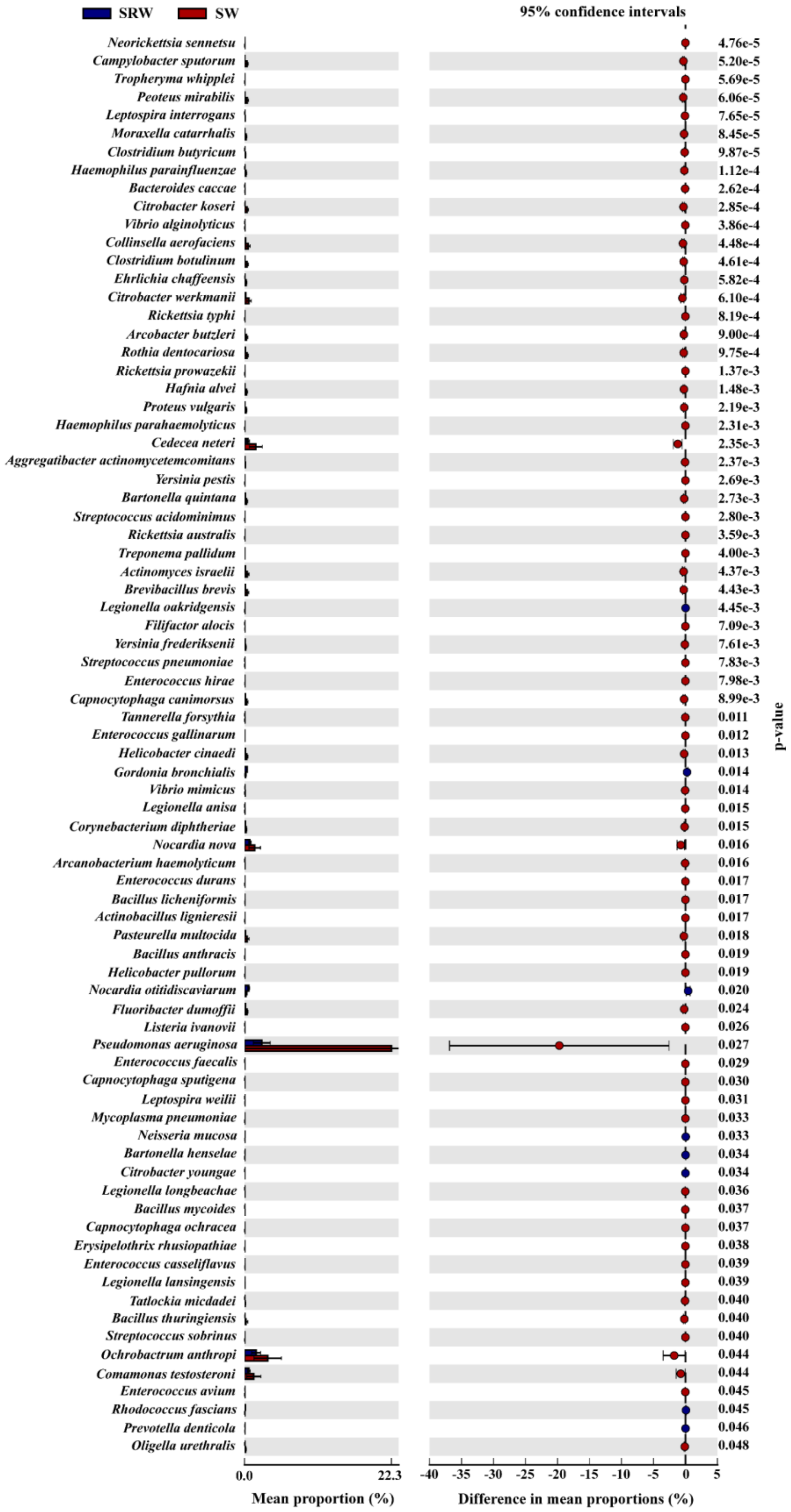


**Figure S2** Extended error bar plot showing pathogens with significant differences ($p < 0.05$) under different water supply scenarios. SW: stable water supply samples; SRW: suspension-restoration water supply samples.

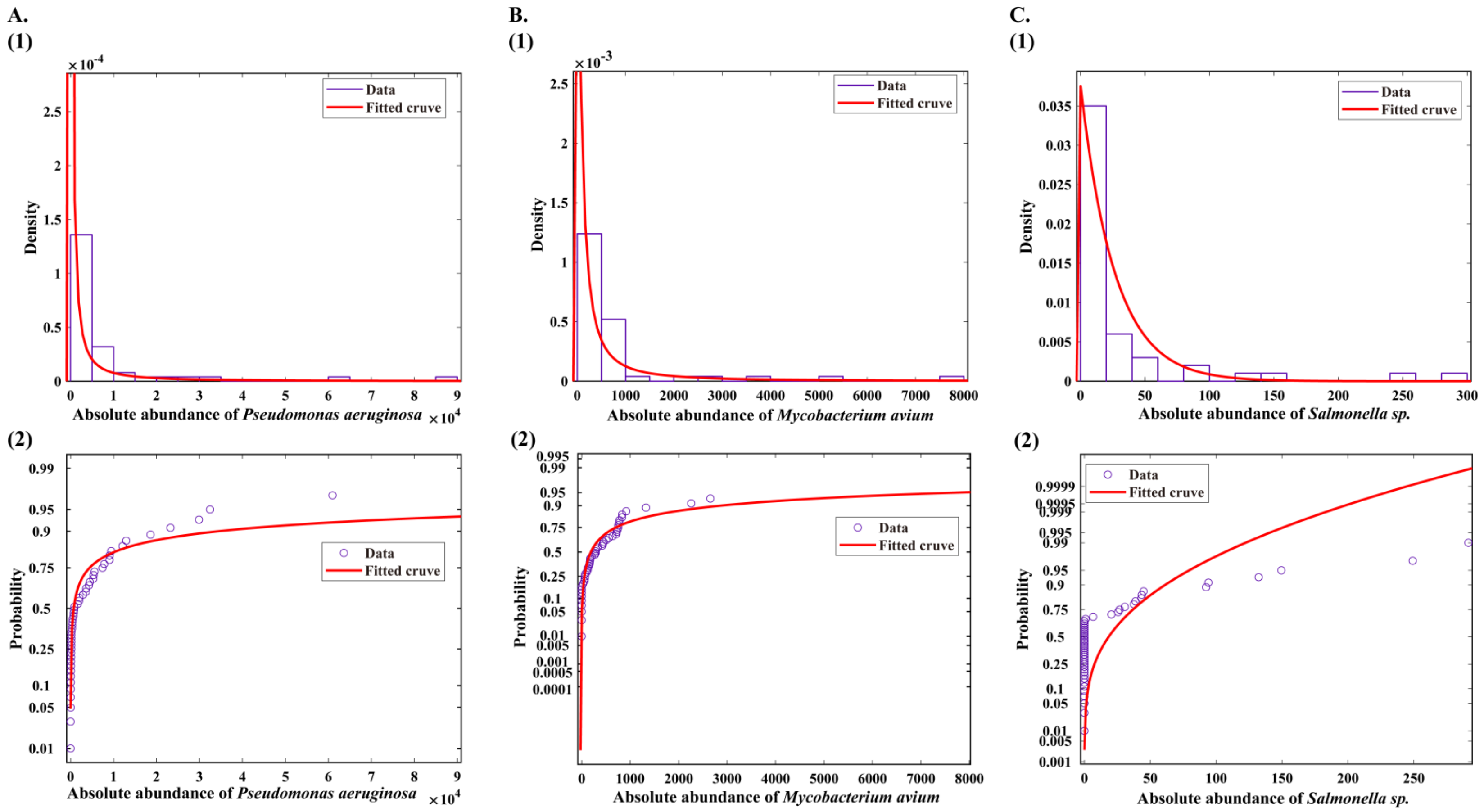


**Figure S3** Probability density distribution diagram (1) and Probability Plot (2) of absolute abundance of the three pathogens. (A. *Pseudomonas aeruginosa*; B. *Mycobacterium avium*; C. *Salmonella* sp.).

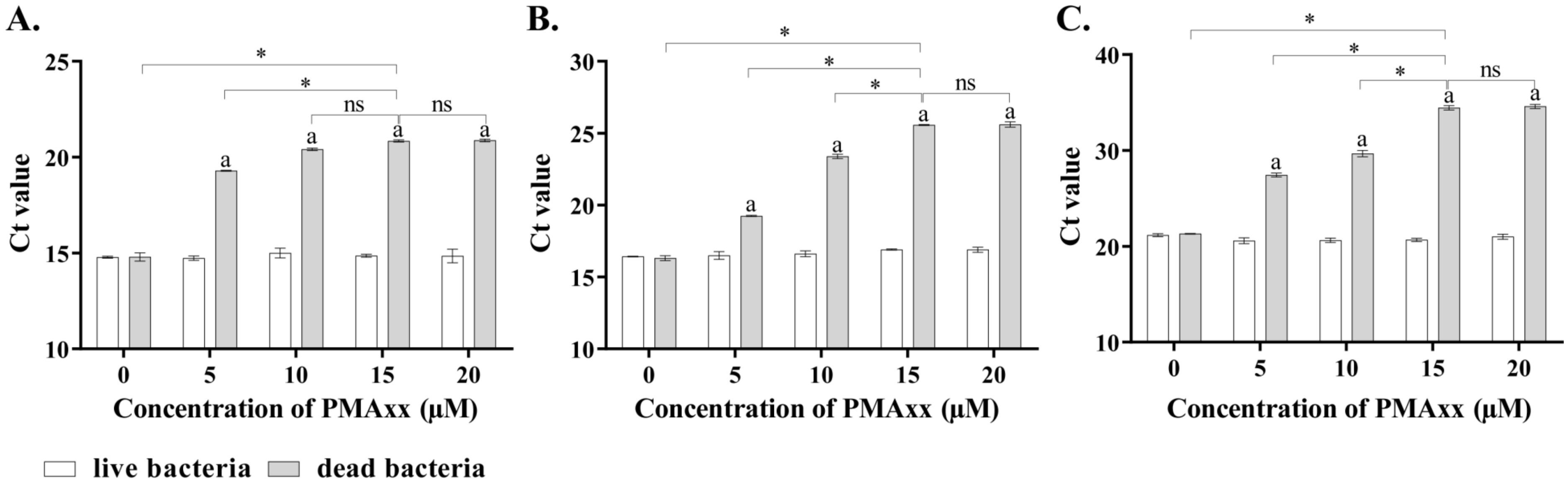


**Figure S4** Histogram showing Ct values of live and dead bacteria under PMAxx treatment with different concentrations. "*" means $p < 0.05$. "a" means significant difference between live and dead bacteria. (A: *Pseudomonas aeruginosa*, B: *Mycobacterium avium*, C: *Salmonella* sp.).

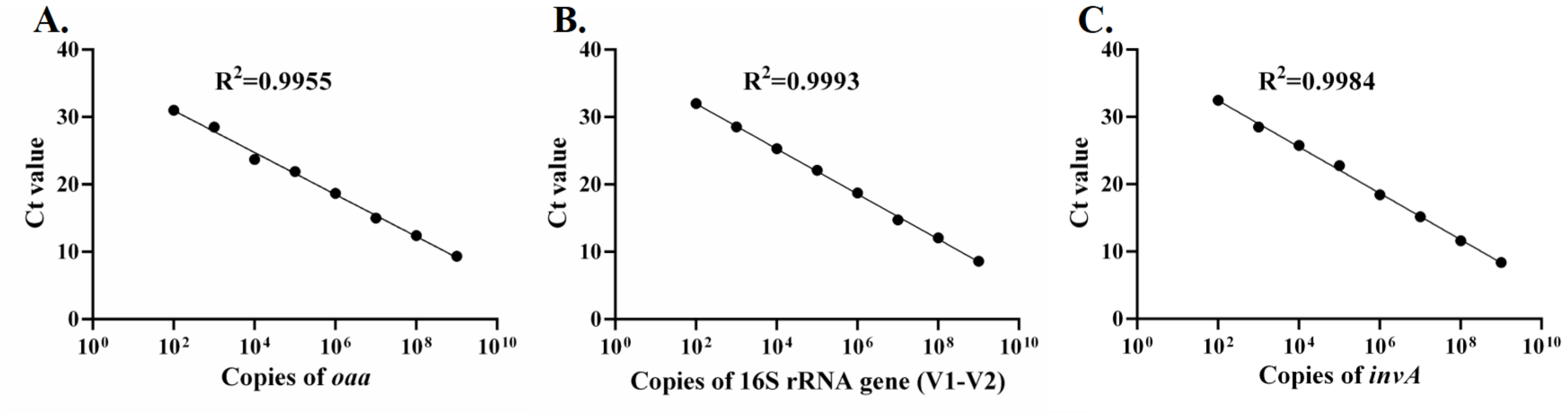


**Figure S5** The standard curve (mean ± standard error) of the three specific single copy genes at the optimal annealing temperature. The amplification efficiencies were 109.9% (A), 98.90% (B) and 95.18% (C).

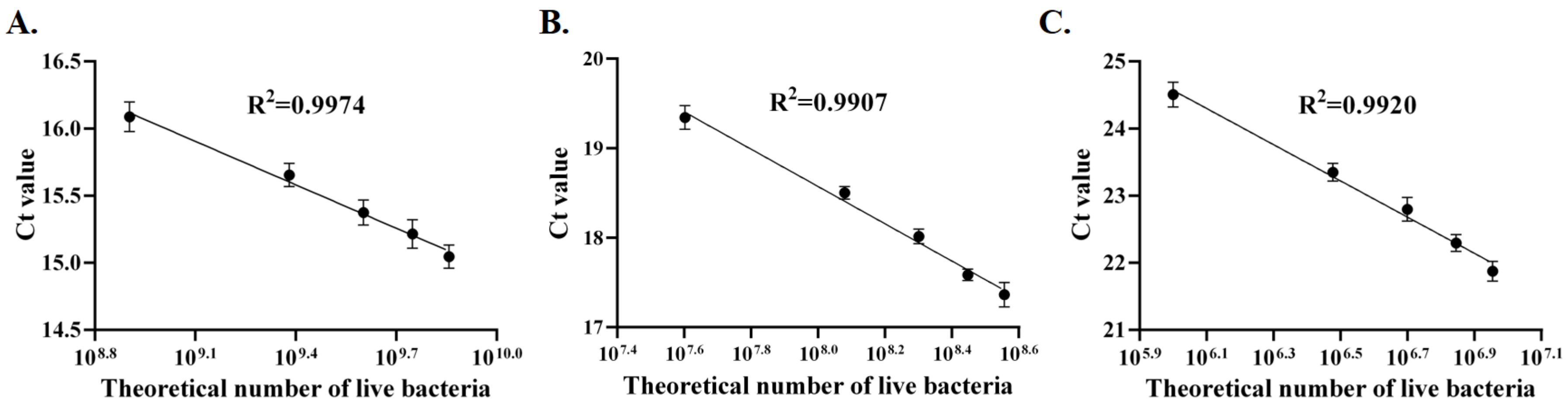


**Figure S6** Correlation between Ct value (mean ± standard error) of live bacteria determined by PMA-qPCR and theoretical number of live bacteria. The Ct value was correlated well with the theoretical number of live bacteria at 10% – 90% (each $R^2 > 0.99$), which indicated that the utilization of PMAxx pretreatment at 15 μM could accurately quantify the three live pathogens in drinking water with good stability. (A. *Pseudomonas aeruginosa*; B. *Mycobacterium avium*; C. *Salmonella* sp.).

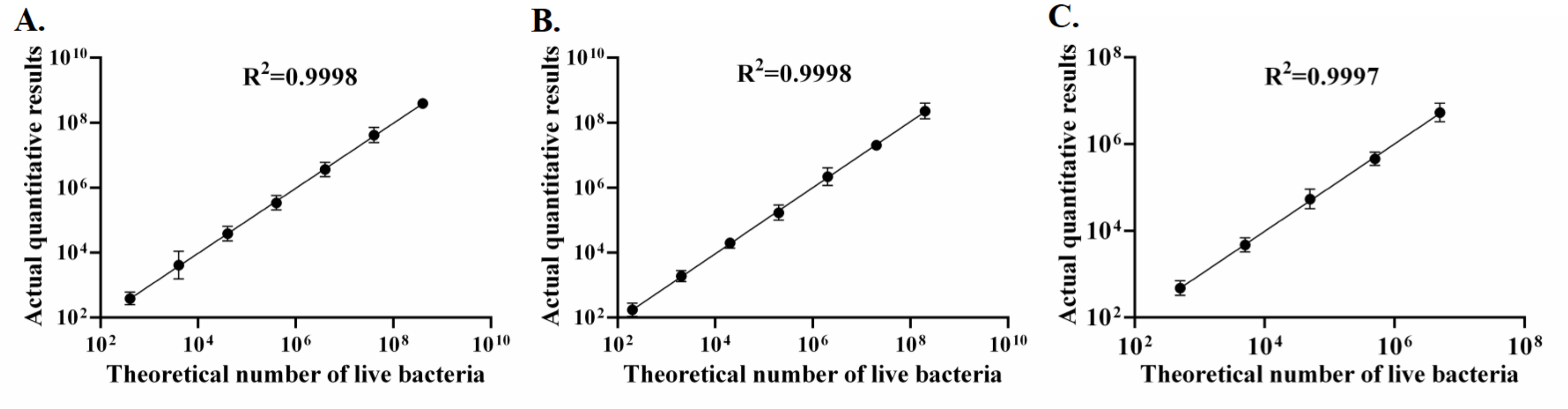


**Figure S7** Linear relationship between the theoretical live count of three pathogens and the actual quantitative results of PMA-qPCR (mean ± standard error). The actual quantitative results were of the same order of magnitude to the theoretical number of live bacteria and appeared a good linear relationship ($R^2 > 0.99$), with an absolute margin of error of less than 10%. (A. *Pseudomonas aeruginosa*; B. *Mycobacterium avium*; C. *Salmonella* sp.).

## References


1 Hamouda, M. A. *et al.* Scenario-based quantitative microbial risk assessment to evaluate the robustness of a drinking water treatment plant. *Water Qual. Res. J. Canada* **51**, 81-96, doi:10.2166/wqrjc.2016.034 (2016).

2 WHO. *Principles and guidelines for the conduct of microbiological risk assessment.* (WHO Press, Geneva, Switzerland, 1999).

3 Falkinham, J. O., Hilborn, E. D., Arduino, M. J., Pruden, A. & Edwards, M. A. Epidemiology and ecology of opportunistic premise plumbing pathogens: *Legionella pneumophila*, *Mycobacterium avium*, and *Pseudomonas aeruginosa*. *Environ. Health Perspect.* **123**, 749-758, doi:10.1289/ehp.1408692 (2015).

4 McClung, R. P. *et al.* Waterborne disease outbreaks associated with environmental and undetermined exposures to water - United States, 2013-2014. *Am. J. Transplant* **18**, 262-267, doi:10.1111/ajt.14607 (2018).

5 Uwamino, Y. *et al.* Showering is associated with *Mycobacterium avium* complex lung disease: An observational study in Japanese women. *J. Infect. Chemother.* **26**, 211-214, doi:10.1016/j.jiac.2019.09.003 (2020).

6 Inderlied, C. B., Kemper, C. A. & Bermudez, L. E. M. The *Mycobacterium avium* complex. *Clin. Microbiol. Rev.* **6**, 266-310, doi:10.1128/cmr.6.3.266-310.1993 (1993).

7 Kirk, M. D. *et al.* World Health Organization estimates of the global and regional disease burden of 22 foodborne bacterial, protozoal, and viral diseases, 2010: A data synthesis. *Plos Med.* **12**, e1001921, doi:10.1371/journal.pmed.1001921 (2015).

8 Stanaway, J. D. *et al.* The global burden of typhoid and paratyphoid fevers: a systematic analysis for the Global Burden of Disease Study 2017. *Lancet Infect. Dis* **19**, 369-381, doi:10.1016/s1473-3099(18)30685-6 (2019).

9 Fewtrell, L. & Bartram, J. *Water quality : guidelines, standards and health : assessment of risk and risk management for water-related infectious diseases / edited by Lorna Fewtrell and Jamie Bartram.*, (World Health Organization., 2001).

10 de Man, H. *et al.* Quantitative assessment of infection risk from exposure to waterborne pathogens in urban floodwater. *Water Res.* **48**, 90-99, doi:10.1016/j.watres.2013.09.022 (2014).

11 Lawinbrussel, C. A., Refojo, M. F., Leong, F. L., Hanninen, L. & Kenyon, K. R. Effect of *Pseudomonas aeruginosa* concentration in experimental contact lens-related microbial keratitis. *Cornea* **12**, 10-18, doi:10.1097/00003226-199301000-00003 (1993).

12 Yangco, B. G., Lackmansmith, C., Espinoza, C. G., Solomon, D. A. & Deresinski, S. C. The hamster model of chronic *Mycobacterium avium* complex infection. *J. Infect. Dis.* **159**, 556-561, doi:10.1093/infdis/159.3.556 (1989).

13 Hornick, R. B. *et al.* Typhoid fever: pathogenesis and immunologic control .1. *N. Engl. J. Med.* **283**, 686-691, doi:10.1056/nejm197009242831306 (1970).
